# Esterified Cholesterol Conjugates Enable LNP‐Mediated mRNA Delivery to the Blood–Brain Barrier Following Intravenous Administration

**DOI:** 10.1002/adma.74762

**Published:** 2026-08-23

**Authors:** Zeru Tian, Xu Wang, Alexis Pacheco Benitez, Erick Guerrero, Yaou Duan, Jingwen Wei, Lukas Farbiak, Daniel J. Siegwart

**Affiliations:** ^1^ Department of Biomedical Engineering, Department of Biochemistry, Simmons Comprehensive Cancer Center, Program in Genetic Drug Engineering University of Texas Southwestern Medical Center Dallas Texas USA

**Keywords:** blood–brain barrier (BBB), brain endothelial cells, cholesterol‐based lipid, lipid nanoparticles (LNPS), mRNA delivery, systemic administration

## Abstract

The blood–brain barrier (BBB) plays a critical role in maintaining central nervous system homeostasis by regulating molecular and cellular exchange between the brain and peripheral circulation. While most delivery strategies focus on crossing the BBB, targeting brain endothelial cells directly may offer a complementary and more controlled alternative for therapeutic intervention. This study presents a ligand‐free lipid nanoparticle (LNP) platform that enables efficient mRNA delivery to the brain endothelium via systemic administration. A modular synthesis strategy was used to generate a chemically diverse library of cholesterol‐based lipids. Screening of 486 LNPs identified an optimized four‐component formulation consisting of C6O2B2 (novel cholesterol‐based lipid), DODAP, DSPC, and DMG‐PEG, which achieves high transfection efficiency in brain endothelial cells without the incorporation of free cholesterol or disruption of BBB integrity and function, such as those utilized in focused ultrasound (FUS) techniques. This BBB‐targeting LNP platform avoids neuronal and glial transfection, thereby offering a noninvasive, and scalable approach for cerebrovascular gene delivery. The work establishes a new class of cholesterol‐based lipids and provides a foundation for next‐generation RNA therapeutics targeting BBB‐associated diseases.

## Graphical abstract

A cholesterol‐conjugated lipid library enabled the identification of a ligand‐free LNP platform for efficient mRNA delivery to brain endothelial cells via systemic administration. This platform achieves selective BBB targeting without disrupting barrier integrity, and enables modulation of neuroinflammation and vascular function without requiring trans‐BBB transport, offering a noninvasive and potentially complementary strategy for cerebrovascular gene modulation.

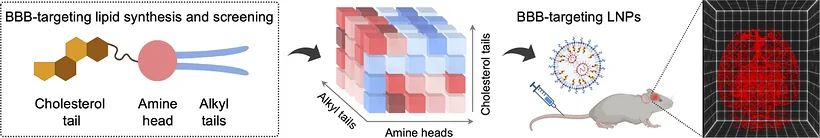

## 1 Introduction

The blood–brain barrier (BBB) is a unique and highly selective boundary between the central nervous system (CNS) and the peripheral vascular circulation [1]. Comprised primarily of brain microvascular endothelial cells interconnected by tight junctions, the BBB maintains brain homeostasis by strictly controlling the movement of ions, nutrients, macromolecules, and immune cells between the blood and brain parenchyma [2]. This essential barrier not only protects the brain from circulating pathogens and toxins, but also ensures proper neuronal function. Recent years have witnessed increasing research focused on strategies to traverse the BBB, including receptor‐mediated transcytosis [3, 4], focused ultrasound [5, 6], and engineered nanoparticles as BBB transport systems via invasive or noninvasive administration [2, 7]. These innovations and progress have fueled great excitement for treatment of neurological diseases; however, crossing the BBB efficiently and selectively still remains challenging, and is often limited by low delivery efficiency, poor cell specificity, or the need for barrier disruption which risks adverse side effects such as hemorrhage and tissue damage. Moreover, delivery across the BBB may not always be necessary—or even desirable—depending on the indication and therapeutic context.

It has often been ignored that the BBB itself is not merely a passive barrier, but an inherently unique and valuable therapeutic target for a litany of therapeutic indications [2]. As the principal cellular components of the BBB, brain endothelial cells regulate neurovascular coupling, immune surveillance, and communication between the peripheral and central immune systems [8, 9, 10]. Dysfunction of these endothelial cells has been implicated in a range of neurological pathologies and chronic CNS disorders, including Alzheimer's disease, stroke, multiple sclerosis, brain metastases, and neuroinflammation [11, 12, 13]. From a translational perspective, strategies that target the BBB without disrupting it offer an alternative and more controlled targeting approach. In many therapeutic applications, it is neither necessary nor ideal to cross the BBB and expose neurons and glia to circulating agents. For instance, modulating endothelial transporter expression, delivering localized anti‐inflammatory signals, or expressing therapeutic proteins at the luminal side of the BBB can have profound effects on CNS health without the associated risks of entering the brain parenchyma [14, 15]. In these cases, such approaches help restore vascular function, support the neurovascular unit, and reduce neuroinflammation, while minimizing the risk of off‐target effects within the brain.

Despite its promise, targeting BBB directly remains underexplored due to the lack of efficient and selective delivery systems, with only a few advanced studies reported [14, 15, 16, 17]—most of which rely on active targeting mechanisms such as antibody or ligand conjugation, which increase formulation complexity and risk of immunogenicity. Therefore, a ligand‐free delivery system that achieves selective BBB‐targeting via noninvasive systemic administration would represent a significant advancement for BBB‐focused therapies. Currently, lipid nanoparticles (LNPs) delivering messenger RNA (mRNA) therapeutics have emerged as a leading modality in genetic medicines as they provide an effective and scalable approach for clinical translation across a diverse variety of disease indications [18, 19, 20, 21, 22, 23]. While LNPs have been proven as highly efficient vehicles for hepatic delivery, expanding their application to other tissues, especially the brain, remains a significant challenge. Conventional LNPs typically consist of four components: an ionizable lipid, cholesterol, a helper lipid, and a polyethylene glycol (PEG) lipid. Among them, cholesterol plays a crucial role in stabilizing the lipid bilayer, enhancing membrane fusion, and facilitating intracellular delivery of mRNA. It is known that the molecular structure of cholesterol derivatives within LNPs can further affect their delivery efficiency and biodistribution [22, 24]. Such cholesterol modifications offer a promising space to fine‐tune LNP targeting without relying on external ligands or complex formulation strategies.

In this study, we report the development of an LNP platform that enables efficient mRNA delivery to the brain vasculature and endothelial cells following intravenous (I.V.) administration. To achieve this, we designed a modular one‐pot, two‐step, three‐component synthesis strategy to construct a chemically diverse library of cholesterol‐based lipids with varied amine headgroups, alkyl tails, and cholesterol moieties. Through systematic in vitro and in vivo evaluation of 486 LNPs, we identified an optimized formulation consisting of C6O2B2, DODAP, DSPC and DMG‐PEG at a molar ratio of 35:50:30:2, which demonstrated the highest BBB‐targeting efficiency without the inclusion of free cholesterol. This ligand‐free, four‐component system achieved efficient delivery to the overall brain vasculatures, particularly the brain endothelial cells, while preserving BBB integrity, thereby supporting its potential for safe therapeutic application. Mechanistic studies revealed markedly improved membrane fusion and enhanced cellular uptake in brain endothelial cells when compared with conventional LNPs. Protein corona analyses of the LNPs further suggested that cholesterol conjugation altered surface protein adsorption profiles, which may contribute to the endogenous BBB specificity. Overall, our work establishes a simple, noninvasive delivery system for brain vasculature by introducing novel cholesterol‐based lipid design, providing a promising strategy for efficient and safe BBB‐targeting LNPs, and opening new opportunities for material‐driven approaches to vascular gene modulation in CNS therapeutics.

## 2 Results and Discussion

### 2.1 One‐Pot Synthesis Strategy Generates 51 BBB‐Targeting Lipids With Tunable Properties

We developed a modular, one‐pot, two‐step, three‐component synthesis strategy that enables efficient, high‐yield, and parallel construction of a chemically diverse library of lipids for exploratory high‐throughput screening of potential BBB delivery composed of varied amine headgroups, alkyl tails, and cholesterol moieties (Figure 1a). This design was motivated by our initial observation that a small subset of cholesterol‐conjugated ionizable lipids exhibited preferential delivery to the brain endothelium. Based on this discovery, we hypothesized that specific structural features contribute to BBB‐associated targeting and subsequently rationally designed a broader library to systematically investigate and optimize these properties. The synthesis begins with either the formation of synthesized core structures through the conjugation of amine heads (C1‐C6) with alkyl acrylates (O1, O2), or by directly utilizing commercially available cores such as SM‐102, DLin‐DMA‐MC3, and many more (Figure 1b; Figures S1–S7). These tertiary amino lipid cores were subsequently functionalized with cholesterol‐derived alkyl bromides to generate a cholesterol‐based quaternary amino lipid structure. This approach yielded a library of 51 new lipids featuring three key elements: (i) incorporation of both quaternary and tertiary amines within a single lipid molecule to fine‐tune lipid ionization, thereby enabling buffering of the acidic endosomal environment and promoting endosomal escape; (ii) inclusion of a degradable ester linkage between the alkyl chain and the amine to enhance biocompatibility, and (iii) tunable spacing between the cholesterol group and the amine core using 3‐, 5‐, or 7‐carbon linkers to improve the cholesterol flexibility and structural adaptability. This catalyst‐free and high‐efficiency synthesis eliminates the need for protection and deprotection steps, and obtains lipids as the exclusive products with overall yields of >80%, offering a simple and scalable route for generating structurally diverse lipid libraries.

**FIGURE 1:**
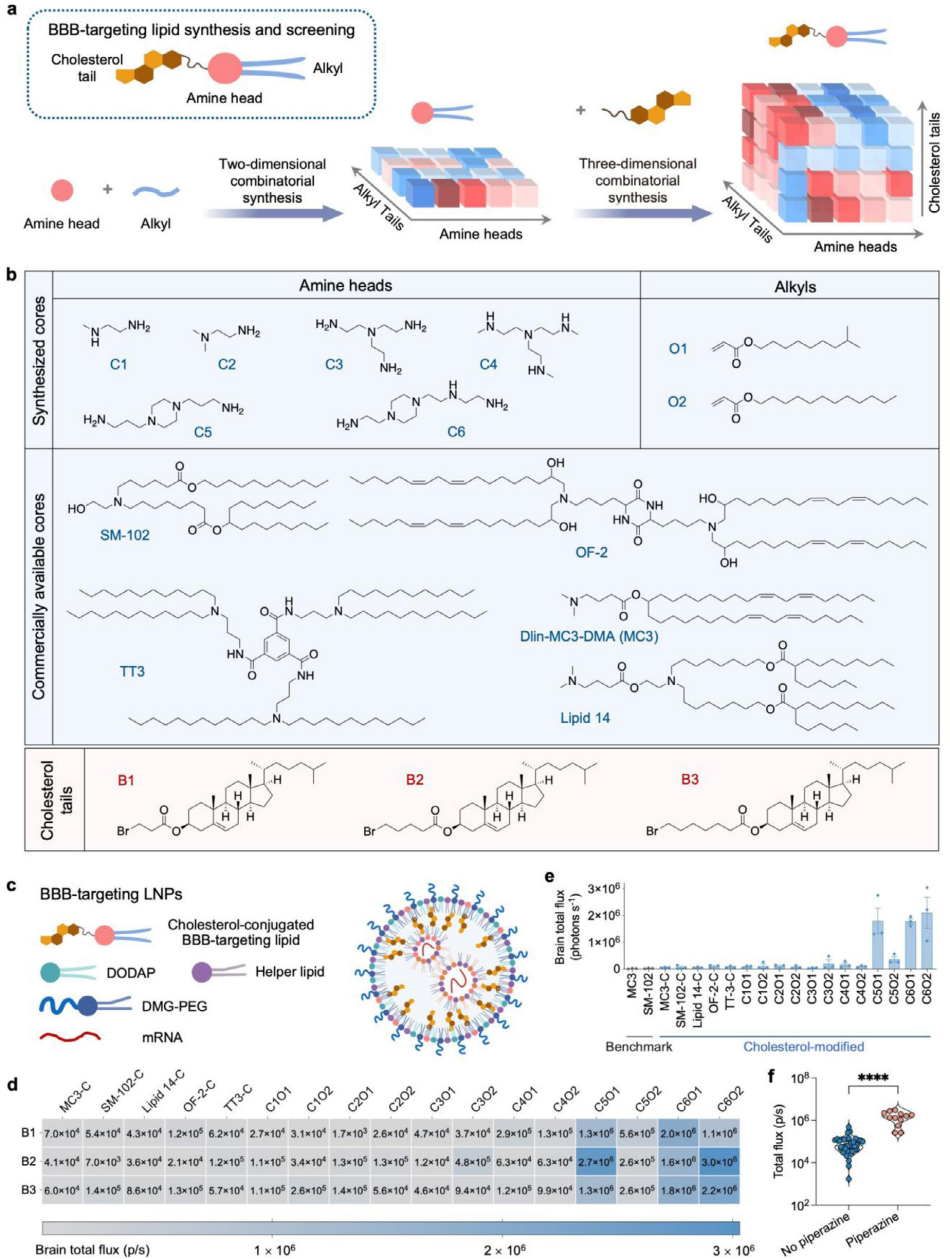
A three‐dimensional (3D) combinatorial synthesis approach was used to develop and evaluate lipids for mRNA delivery to the BBB. (a) Schematic representation of the 3D combinatorial synthesis strategy employed to identify BBB‐targeting lipids. (b) Chemical structures of the lipid library components, including amine head groups (C1–C6), alkyl groups (O1 and O2), and cholesterol tails (B1, B2, and B3). (c) Schematic illustration of mRNA‐encapsulating lipid nanoparticles (LNPs). (d) Heatmap showing the quantification of total luciferase activity in the brain following systemic administration of BBB‐targeting LNPs (*n* = 3 biologically independent samples). (e) Total brain luciferase activity mediated by BBB‐targeting LNPs formulated with cholesterol‐conjugated lipids (synthesized core structures are shown), and benchmark MC3 and SM‐102 LNPs formulated with unmodified ionizable lipids. (f) Impact of piperazine incorporation in the lipid core on BBB‐targeting efficacy, demonstrating significantly enhanced activity in piperazine‐containing formulations (****p < 0.0001).

### 2.2 C6O2B2 Identified as the Lead Lipid for BBB‐Targeted mRNA Delivery

To evaluate the mRNA delivery potential of the designed lipid library derived from initial BBB‐targeting leads, each lipid was incorporated into LNPs by replacing free cholesterol in a formulation containing 1,2‐dioleoyl‐3‐dimethylammonium‐propane (DODAP), 1,2‐dioleoyl‐*sn*‐glycero‐3‐phosphoethanolamine (DOPE), and 1,2‐dimyristoyl‐*rac*‐glycero‐3‐methoxypolyethylene glycol‐2000 (DMG‐PEG) at a molar ratio of 47.6/23.8/23.8/4.8 (cholesterol‐conjugated BBB‐targeting lipid/DODAP/DOPE/DMG‐PEG), following our previously established mRNA‐optimized mDLNP formulation using the ethanol dilution method (Figure 1c) [25, 26]. Firefly luciferase (Luc) mRNA was encapsulated in the LNPs, and the brain luciferase activity was quantified after systemic administration (Figure 1d). As shown in Figure 1d,e, conventional MC3 LNPs and SM‐102 LNPs formulated with unmodified ionizable lipids exhibited minimal brain delivery following intravenous administration, whereas BBB‐targeting LNPs achieved substantially higher brain transfection. The results revealed that while variations in the alkyl and cholesterol tails had minimal impact on BBB‐targeting efficiency (Figure S8), the structure of the amino lipid core was critical, with piperazine‐containing lipids demonstrating significantly enhanced delivery compared with non‐piperazine cores (Figure 1d–f). Consistently, hit rate analysis further showed a higher proportion of effective formulations among piperazine‐containing lipids, with the C6 piperazine core exhibiting superior performance (Figure S9). Membrane fusion analysis showed that variations in alkyl chains and cholesterol‐derived tails had minimal impact, whereas the piperazine core played a dominant role, with C6‐based lipids, particularly C6O2B2, exhibiting the highest capability (Figure S10). Among all candidates, C6O2B2 exhibited the highest brain delivery efficiency and was therefore selected for further investigation.

### 2.3 High‐Throughput Screening Identifies an Optimized LNP for BBB‐Targeted mRNA Delivery

Helper lipids and overall LNP composition also play a critical role in determining mRNA delivery efficiency, endosomal escape, and tissue distribution. To further optimize the BBB‐targeting LNP system, we selected C6O2B2 as a representative BBB‐targeting lipid (Figure 2a) and formulated it with ionizable lipid DODAP, the PEGylated component DMG‐PEG2000, and different phospholipids, all at varying lipid molar ratios. The helper phospholipids represent a diverse set of headgroup chemistries, including DOPE, 1,2‐distearoyl‐*sn*‐glycero‐3‐phosphocholine (DSPC), 1,2‐dioleoyl‐*sn*‐glycero‐3‐phosphocholine (DOPC), 1,2‐dimyristoyl‐*sn*‐glycero‐3‐phosphocholine (DMPC), 1,2‐dioleoyl‐*sn*‐glycero‐3‐phospho‐(1'‐*rac*‐glycerol) (sodium salt) (DOPG), and sphingomyelin (EggSM). To elucidate the optimal formulation parameters, the relative molar ratios of lipids within LNPs were adjusted across the following ranges: C6O2B2 (35, 50, 65), DODAP (10, 30, 50), helper phospholipid (10, 30, 50), and DMG‐PEG (1, 2, 4), yielding a library of 486 C6O2B2 LNP formulations for in vitro and in vivo evaluation of mRNA delivery performance (Figure 2b).

**FIGURE 2:**
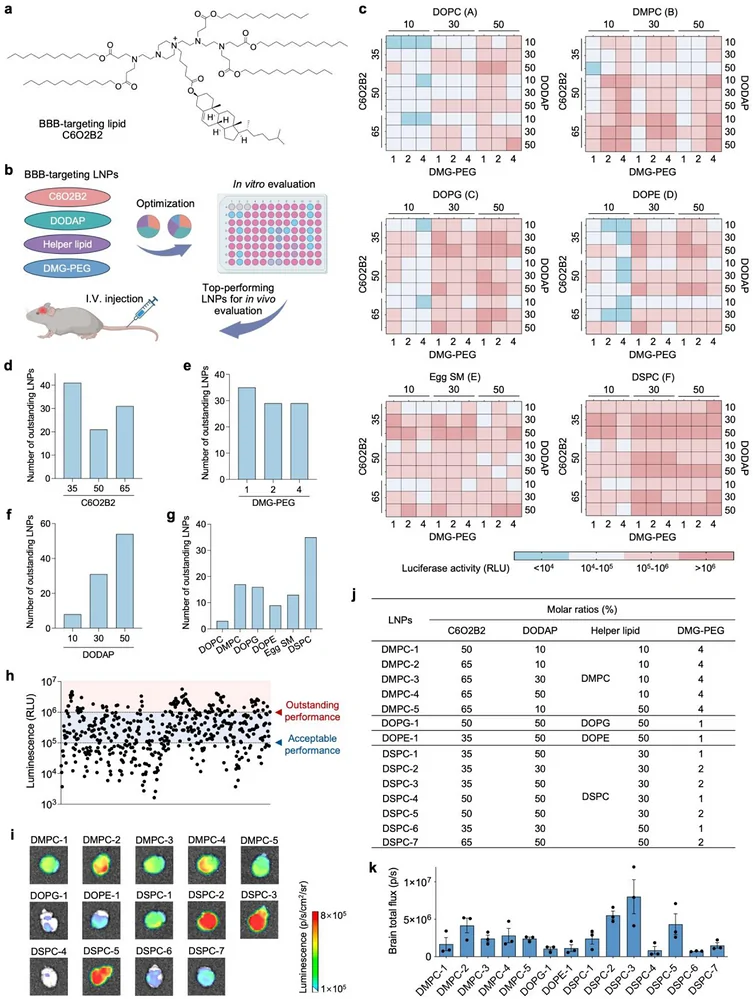
High‐throughput screening and in vivo validation of BBB‐targeting LNP formulations. (a) Chemical structure of the top‐performing BBB‐targeting lipid C6O2B2. (b) Schematic of the screening pipeline. A total of 486 LNP formulations with varying helper lipids and component ratios were systematically evaluated for in vitro transfection efficiency. The top‐performing candidates were then assessed in vivo via intravenous (I.V.) administration of luciferase mRNA‐loaded LNPs (0.1 mg/kg, 6 h) in C57BL/6 mice. (c) High‐throughput screening of LNP transfection efficiency in IGROV‐1 cells. Luciferase mRNA (mLuc)‐loaded LNPs were prepared by pipette mixing and used to transfect IGROV‐1 cells (20 ng mRNA per well, 24 h; *n* = 3 biologically independent samples). Heat map displays relative luciferase expression, with relative light units (RLU) >1 000 000 considered as highly efficient. (d–f) Relative hit rate of BBB‐targeting LNPs at different molar ratios of (d) C6O2B2, (e) DMG‐PEG, and (f) DODAP. (g) Hit rate of LNPs incorporating different helper lipids. (h) Performance classification of screened LNPs based on luminescence: >1 000 000 RLU (highly efficient), 100 000–1 000 000 RLU (moderate efficiency). (i–k) Bioluminescence imaging (BLI) was performed 6 h post‐intravenous administration of mLuc‐loaded LNPs (0.1 mg/kg), and total brain luminescence flux was quantified (*n* = 3). (i) Representative bioluminescence images of the brain for the top 14 LNP formulations. The images were selected from mice with signal intensities consistent with the group average and represent the overall trends observed across all animals. (j) Molar composition of the top 14 formulations. (k) Quantification of brain luminescence signals across the top‐performing formulations. Each column was compared with DSPC‐3 formulation using one‐way ANOVA with Tukey's multiple comparisons test. Statistical significance is indicated above each column. Data are presented as mean ± SEM. *p < 0.05; **p < 0.01; ***p < 0.001; ****p < 0.0001; ns, not significant, *p* > 0.05.

We first evaluated the delivery efficiency of all LNP formulations using Luc mRNA by measuring luciferase expression in IGROV1 cells, a representative cell line with moderate transfection resistance commonly used as a standard for in vitro mRNA delivery assessment (Figure 2c). LNPs achieving relative luminescence units (RLU) > 10^6^ and > 10^5^ were considered as “outstanding” and “acceptable”, respectively. We observed that variations in DMG‐PEG content had minimal impact on mRNA delivery efficiency, whereas modulation of the other three components substantially influenced luciferase expression levels (Figure 2d–g; Figure S11). LNPs containing less cholesterol‐conjugated BBB‐targeting lipid C6O2B2 appeared more effective, while increased ionizable DODAP content demonstrated a positive correlation with delivery efficiency. Among the six tested helper phospholipids, DSPC mediated the highest luciferase activity (Figure S11). Out of 486 LNP formulations, 240 met the threshold for “acceptable” performance, with 91 LNPs categorized as “outstanding” (Figure 2h). Most LNPs exhibited low cytotoxicity, and those with relatively high toxicity (cell viability <80%) were excluded from further analysis (Figure S12). We then selected the 14 top‐performing LNPs for further in vivo studies (Figure 2j,i; Table S1). Of these, the DSPC‐3 formulation (C6O2B2/DODAP/DSPC/DMG‐PEG at a molar ratio of 35/50/30/2) achieved the highest luminescence signal in mouse brain after systemic administration (Figure 2i,k). This indicates that DSPC‐3 most effectively mediates brain delivery of mRNA among the LNPs evaluated. The robust in vivo signal underscores its potential for BBB‐targeted delivery and highlights the critical role of lipid composition—particularly this specific molar ratio—in modulating biodistribution and enhancing transfection efficiency in the BBB.

### 2.4 BBB‐Targeting LNPs Efficiently Deliver mRNA to BBB Endothelium

Based on these results, LNPs composed of C6O2B2, DODAP, DSPC, and DMG‐PEG at a molar ratio of 35/50/30/2 were selected as the lead candidate for subsequent investigation (Figure 3a). Characterization revealed that the nanoparticles were well‐structured, with a suitable hydrodynamic diameter of ∼138 nm and a low polydispersity index (PDI) of ∼0.14 (Figure 3a,b). The overall LNPs exhibited a near‐neutral, slightly positive surface charge and yielded a high mRNA encapsulation efficiency (EE) of 95% (Figure 3a). These properties demonstrate that the new BBB‐targeting LNPs have a strong ability to condense and encapsulate mRNA molecules, even in the absence of unmodified cholesterol.

**FIGURE 3:**
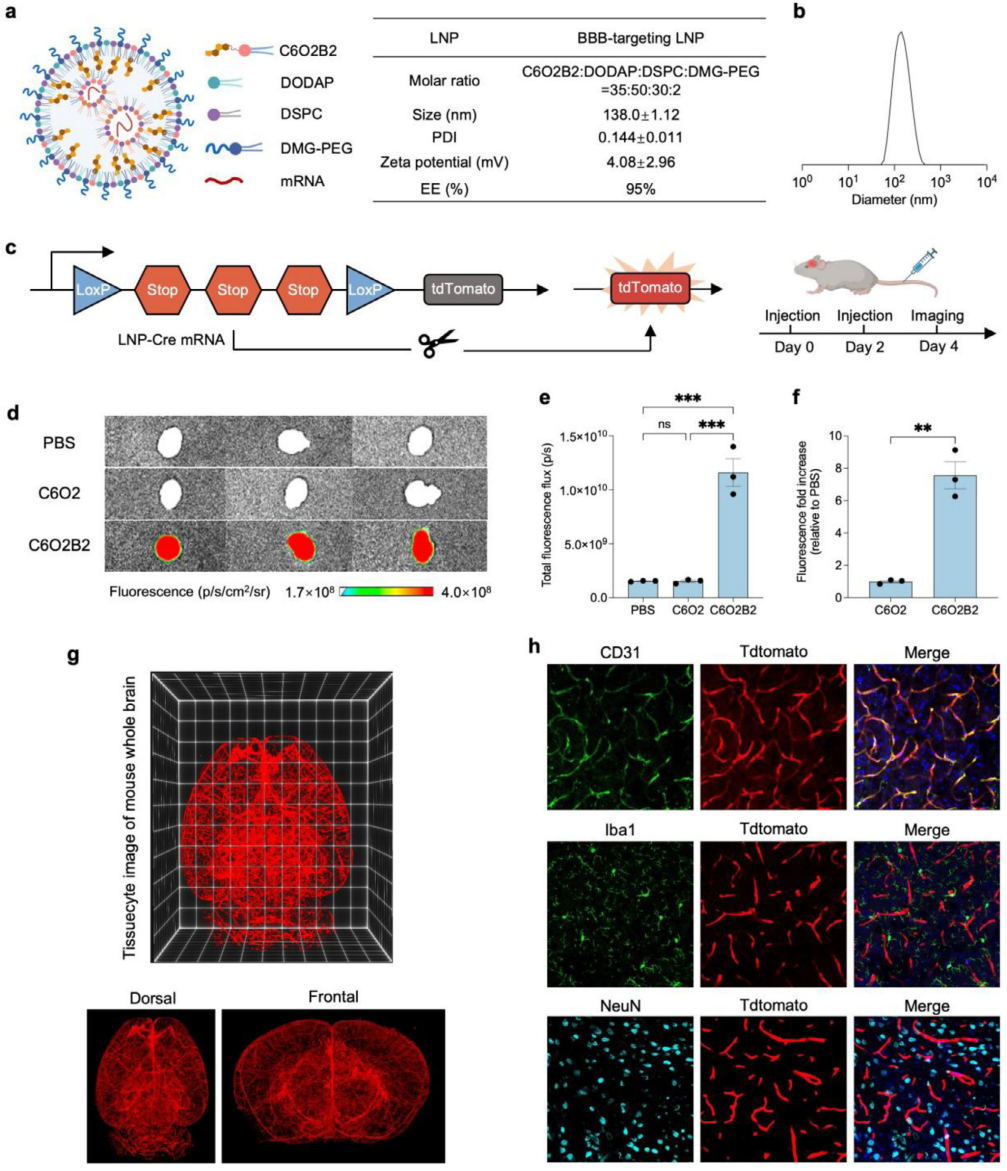
Characterization of BBB‐targeting LNPs and cell type selectivity evaluation of BBB‐targeting. (a) Physicochemical characterization of C6O2B2 LNPs formulated in 10 mM citrate buffer (pH 4.0) (*n* = 3 biologically independent samples). (b) Hydrodynamic diameter distribution of the C6O2B2 LNP. (c) Schematic illustrating Cre mRNA delivery, leading to tdTomato expression in transgenic mice through Cre‐mediated deletion of the stop cassette. (d) Ex vivo fluorescence imaging of brains from tdTomato mice following intravenous injection of C6O2 and C6O2B2 LNPs loaded with Cre mRNA (0.3 mg/kg, two doses, 48 h post‐final injection). (e) C6O2 and C6O2B2 LNPs induced tdTomato expression in the brain following Cre mRNA administration (0.3 mg/kg, two doses). The C6O2B2 LNP formulation exhibited significantly higher tdTomato expression compared to PBS controls. Data are presented as mean ± s.e.m. (*n* = 3 biologically independent animals). (f) The fold increase in brain fluorescence for C6O2 and C6O2B2 LNPs compared to PBS‐treated mice. (g) Representative tissuecyte image of a whole brain from an LNP‐Cre–treated mouse. (h) Confocal images of brain sections showing co‐localization of tdTomato with CD31, Iba1, and NeuN, indicating expression in endothelial cells, microglia, and neurons, respectively.

We next examined their transfection efficiency in specific brain cell types using a genetically engineered Cre‐LoxP mouse model (Ai14). This model harbors LoxP‐flanked “stop” sequences upstream of the tdTomato gene, allowing Cre mRNA delivery and subsequent translation into CRE recombinase protein to induce tdTomato fluorescence upon successful recombination (Figure 3c). As a control, the C6O2 lipid without cholesterol conjugation (Figure S13) was formulated with free cholesterol to produce C6O2 LNPs, using C6O2/cholesterol/DODAP/DSPC/DMG‐PEG at a molar ratio of 35:35:50:30:2. Following two intravenous doses of 0.3 mg/kg Cre mRNA, robust tdTomato expression was observed throughout the brain in LNP‐treated mice, significantly higher than in PBS and C6O2 treated groups (Figure 3d–f). To assess the spatial distribution of transfected cells, we employed Serial Two‐Photon Tomography (STPT), a high‐resolution imaging technique that enables whole‐brain mapping by serial sectioning and 3D reconstruction using TissueCyte 1000 instrumentation. This approach provided an in‐depth visualization of tdTomato‐positive cells across the entire brain. Strikingly, bright and extensive tdTomato fluorescence was detected throughout the brain vasculature, including surface and penetrating arterioles and venules, as well as microvessels and capillaries (primary structures that constitute the BBB), confirming efficient transfection across the brain vascular network following systemic administration (Figure 3g; Figure S14). Immunofluorescence analysis of the brain sections further revealed strong colocalization of tdTomato with CD31^+^ brain endothelial cells, with no detectable signals in NeuN^+^ neurons or IBA1^+^ microglia (Figure 3h; Figure S15). These results provide direct evidence that the BBB‐targeting LNPs exhibit high selectivity for brain endothelium, without off‐target expression in neuronal or glial cells, indicating the potential for precise genetic modulation of the brain vasculature without crossing BBB. Functional Cre mRNA delivery was modest in most peripheral tissues (Figure S16a) with some transfection of heart endothelial cells (Figure S16b), reinforcing preferential delivery to brain endothelium (Figure S17).

To evaluate the role of individual components, we compared LNPs with or without the BBB‐targeting lipid, and LNPs with or without DODAP. The results demonstrated that DODAP is not required for targeting when combined with the BBB‐targeting lipid C6O2B2, but DODAP plays an important role in enhancing delivery efficiency. Moreover, results clearly showed that the BBB‐targeting lipid C6O2B2 is required for enabling brain targeting (Figure S17).

### 2.5 BBB‐Targeting LNPs Preserve BBB Integrity and Function

To determine whether such LNP‐mediated mRNA delivery compromises BBB integrity, we evaluated BBB permeability following systemic administration. C57BL/6 mice were first intravenously injected with BBB‐targeting LNPs encapsulating Cre mRNA, followed by administration of Lung‐SORT LNPs, Liver‐SORT LNPs, or an additional dose of BBB‐targeting LNPs encapsulating Luc mRNA 24 or 48 h later (Figure 4a). The results suggested that pre‐treatment with BBB‐targeting LNPs did not enhance the uptake of either Lung‐ or Liver‐SORT LNPs in the brain, nor did it improve the delivery efficiency of a subsequent dose of BBB‐targeting LNPs (Figure 4b–e). We next assessed the extravasation of Evans blue dye, an established indicator of BBB permeability [27], after administering C57BL/6 mice with Luc mRNA‐loaded BBB‐targeting LNPs (Figure 4f). Minimal to no dye leakage was detected in the brains of mice treated with BBB‐targeting LNPs, indicating intact BBB structure (Figure 4g,h).

**FIGURE 4:**
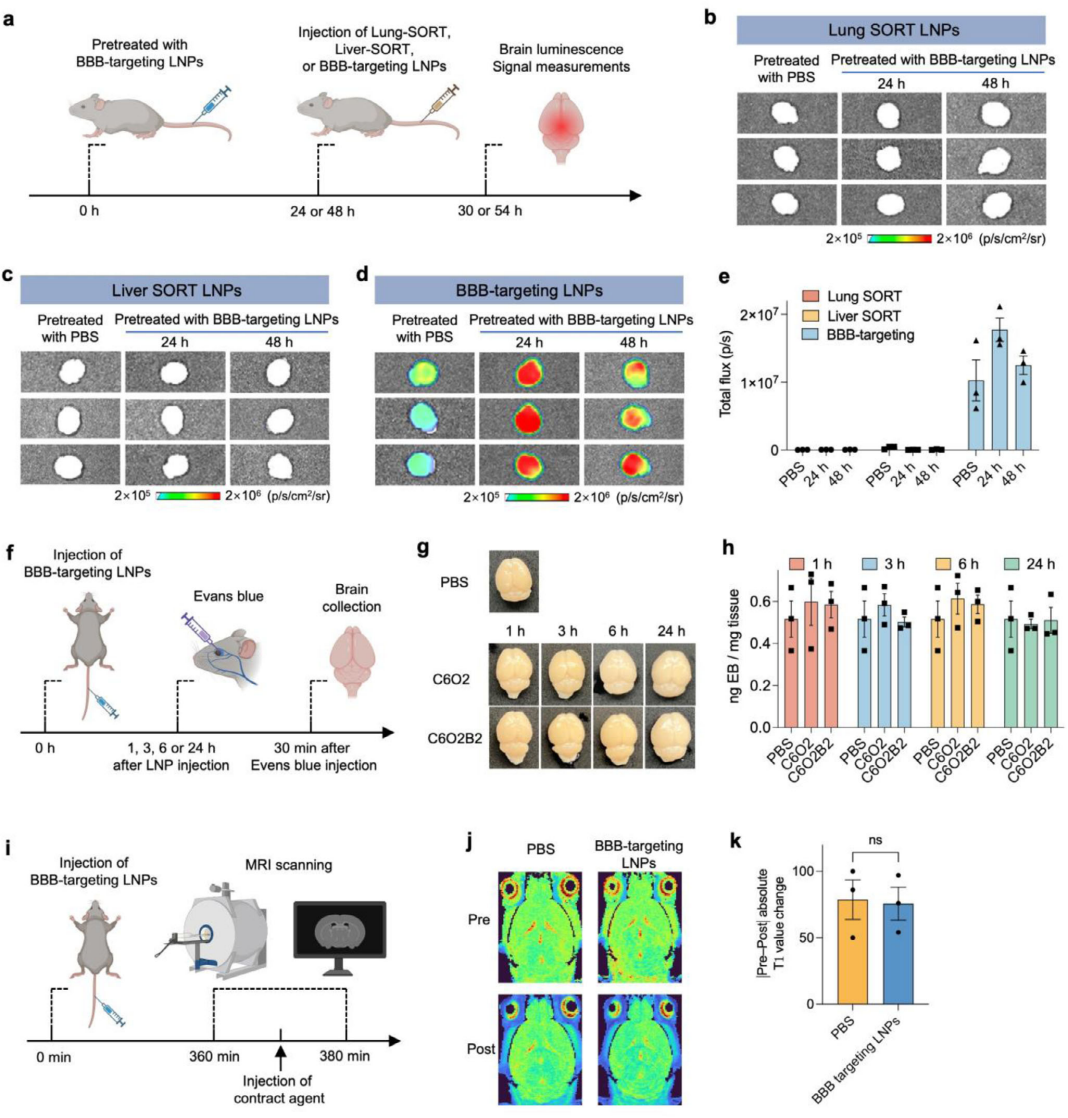
Evaluation of BBB integrity after treatment with BBB‐targeting LNPs. (a) Schematic representation of the experimental setup used to assess the effect of BBB‐targeting lipids on BBB permeability. C57BL/6 mice were intravenously injected with Cre mRNA‐loaded BBB‐targeting LNPs (0.3 mg/kg mRNA). At 24 or 48 h post‐injection, luciferase mRNA‐loaded LNPs (0.1 mg/kg) were administered intravenously (I.V.) and analyzed after 6 h. (b–d) Representative bioluminescence images of the brain following administration of (b) lung‐targeting, (c) liver‐targeting, and (d) BBB‐targeting LNPs in C57BL/6 mice. (e) Quantification of total luciferase activity in the brain after intravenous injection of lung‐, liver‐, and BBB‐targeting LNPs. (f) Schematic of BBB integrity assessment using Evans blue dye. C57BL/6 mice were intravenously injected with mLuc‐loaded C6O2 and C6O2B2 LNPs (0.3 mg/kg mRNA). Evans blue was administered at 1, 3, 6, or 24 h post‐injection, followed by (g) brain collection and imaging and (h) homogenization to quantify Evans blue penetration. (i) Schematic representation of BBB functional evaluation using magnetic resonance imaging (MRI). (j) Representative T1‐weighted images of the brain before and after administration of Gadobutrol (1.0 mmol/kg). (k) Absolute change in T1 values before and after Gadobutrol administration.

In addition, we performed magnetic resonance imaging (MRI) using a 7T small animal MRI spectrometer to assess the potential BBB disruption. A gadolinium‐based contrast agent was employed to the mice 6 h after the intravenous injection of BBB‐targeting LNPs to detect any abnormal vascular permeability (Figure 4i). Comparison of longitudinal relaxation (T1) maps pre‐ and post‐contrast administration showed no contrast enhancement in the brain, confirming no structural or functional BBB alterations under the tested conditions (Figure 4j,k). Together, these results demonstrate that systemic administration of BBB‐targeting LNPs can deliver mRNA across the BBB without compromising its integrity or function, supporting their safety for further therapeutic application.

To assess the therapeutic potential of brain endothelial‐targeted mRNA delivery, IL‐10 mRNA was administered via BBB‐targeting C6O2B2 LNPs in an LPS‐induced acute brain injury model. In both prophylactic and therapeutic settings, C6O2B2 treatment more effectively reduced Evans blue extravasation compared with SM‐102 LNPs and C6O2 LNPs, indicating improved BBB integrity. C6O2B2 treatment also resulted in greater suppression of pro‐inflammatory cytokines, including TNF‐α, IL‐1β, and IL‐6, demonstrating that selective brain endothelial transfection can modulate neuroinflammation and protect vascular function (Figure S18).

### 2.6 Dual Mechanisms Enable BBB‐Targeting LNP Delivery

To elucidate potential mechanisms responsible for the observed BBB‐targeting, we investigated three possibilities: (1) enhanced endosomal escape; (2) indirect pathways, such as immune cell transfection, exosome transport, or receptor‐mediated uptake; and (3) endogenous targeting mediated by protein corona formation.

Efficient endosomal escape is a critical determinant of the effectiveness of LNP‐based RNA delivery [28, 29]. We hypothesized that the cholesterol‐conjugated BBB‐targeting lipid acts as a structurally active component that facilitates enhanced endosomal escape in multiple ways, thereby improving cytosolic mRNA delivery and transfection efficiency. Incorporating both quaternary and tertiary amines within a single lipid molecule enables endosomal pH buffering by modulating lipid ionization, which promotes membrane fusion; the bulky cholesterol moiety alters lipid packing, increasing the distance between alkyl chains through a conical geometry; covalent linkage of cholesterol further anchors the lipid within the LNP and endosomal bilayer to enhance membrane destabilization, neither of which is expected to occur with free cholesterol (Figure 5a). To test this hypothesis, we performed a fluorescence resonance energy transfer (FRET) assay to assess LNP membrane fusion and dissociation. Endosomal‐mimicking liposomes were co‐labeled with the donor fluorophore 1,2‐dioleoyl‐*sn*‐glycero‐3‐phosphoethanolamine‐*N*‐(7‐nitro‐2‐1,3‐benzoxadiazol‐4‐yl) (NBD‐PE) and the acceptor fluorophore 1,2‐dioleoyl‐*sn*‐glycero‐3‐phosphoethanolamine‐*N*‐(lissamine rhodamine B sulfonyl) (Rho‐PE). In intact liposomes, close proximity of the fluorophores results in FRET‐mediated signal quenching, whereas increased donor–acceptor distance results in NBD signal recovery upon membrane fusion (Figure 5b) [30, 31]. Notably, BBB‐targeting LNPs demonstrated rapid fusion with endosomal‐mimicking liposomes upon initial mixing, and the extent of fusion continued to rise over time. These LNPs exhibited significantly higher fusion efficiency than C6O2 LNPs throughout the experiment, indicating a strong capacity to disrupt endosomal membranes (Figure 5c–e). Therefore, we believe that this enhanced fusogenicity may contribute to their superior endosomal escape and efficient cytosolic release of mRNA in target cells.

**FIGURE 5:**
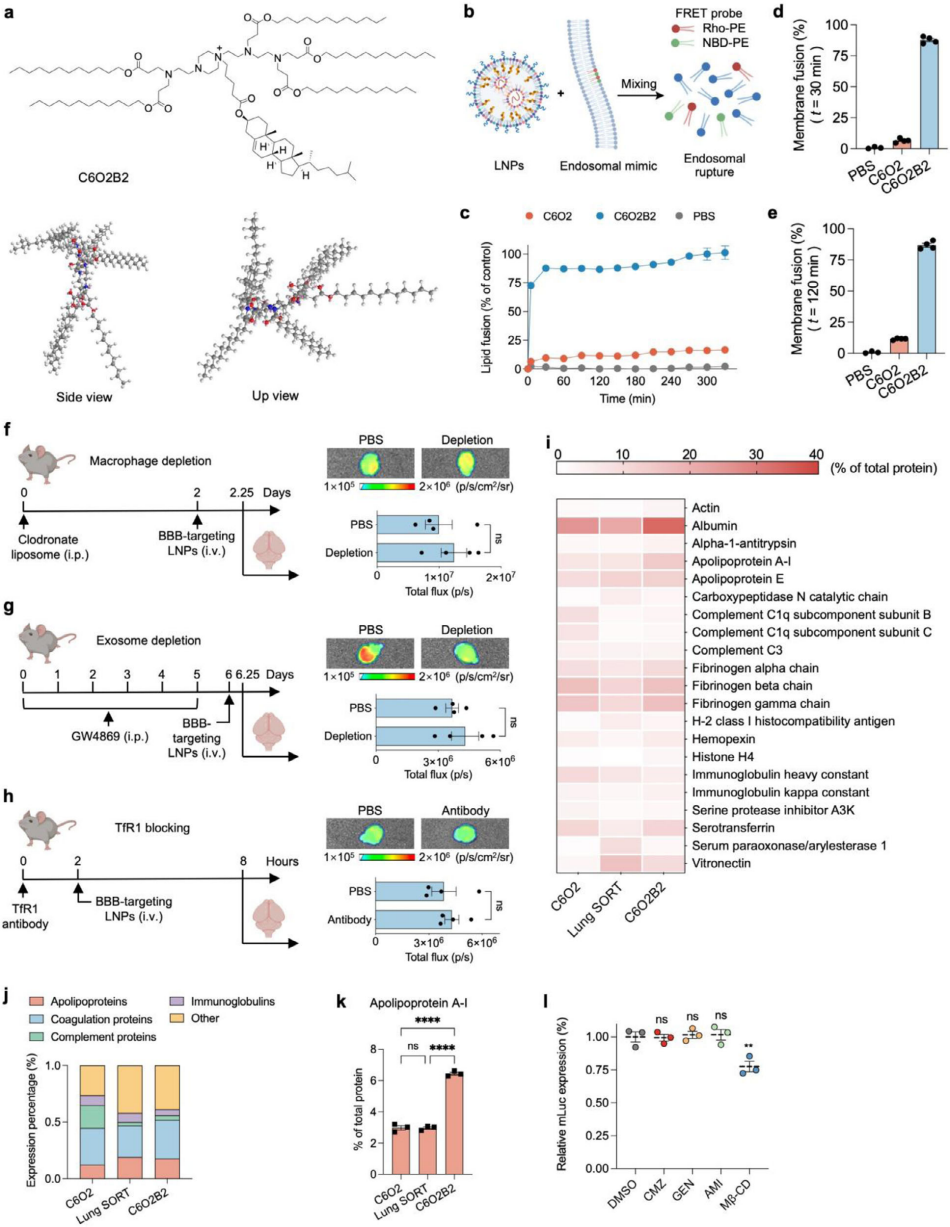
Mechanistic insights into BBB‐targeting LNP delivery. (a) Chemical structure and molecular dynamics simulation of the top‐performing BBB‐targeting lipid C6O2B2, showing side and top views of its predicted conformation. (b) Schematic representation of the study design for assessing the fusogenic properties of BBB‐targeting LNPs using fluorescence resonance energy transfer (FRET). (c) Lipid fusion and membrane destabilization properties of C6O2 and C6O2B2 LNPs, determined by FRET at pH 5.5. (d,e) Quantification of C6O2B2 LNP dissociation via FRET upon mixing with endosome‐mimicking anionic liposomes for 30 min (d) or 120 min (e) at pH 5.5, demonstrating superior lipid fusion efficiency compared to C6O2 LNPs. (f) Evaluation of BBB‐targeting LNP delivery in macrophage‐depleted mice. Mice were intraperitoneally injected with clodronate liposomes (200 µL per mouse) to deplete macrophages, followed by intravenous administration of luciferase mRNA‐loaded LNPs (0.1 mg/kg) 2 days later. Ex vivo bioluminescence imaging and quantification of total brain flux were performed 6 h post‐treatment. (g) Assessment of BBB‐targeting LNP delivery in exosome‐depleted mice. Mice received daily intraperitoneal injections of GW4869 (4 mg/kg) for 5 days to inhibit exosome production, followed by intravenous administration of luciferase mRNA‐loaded LNPs (0.1 mg/kg) on Day 6. Ex vivo bioluminescence imaging and total brain flux quantification were performed 6 h post‐treatment. (h) Evaluation of transferrin receptor 1 (TfR1)‐mediated BBB‐targeting LNP delivery. Mice were intravenously injected with an anti‐TfR1 antibody (4 mg/kg) to block TfR1, followed by administration of luciferase mRNA‐loaded LNPs (0.1 mg/kg) 2 h later. Ex vivo bioluminescence imaging and total brain flux quantification were conducted 6 h post‐treatment. (i) Comparative analysis of protein corona composition among mDLNPs, Lung SORT LNPs, and BBB‐targeting LNPs. (j) Functional classification of the most abundant proteins identified in LNP protein coronas. (k) Quantification of apolipoprotein A‐I enrichment in the protein coronas of BBB‐targeting LNPs compared to C6O2 LNPs and Lung SORT LNPs, highlighting their potential role in facilitating BBB interaction and transport. (l) Inhibition of C6O2B2 LNP uptake by endocytic pathway inhibitors (*n* = 3 biologically independent samples). Uptake of C6O2B2 LNPs was assessed in the presence of specific endocytic inhibitors: chlorpromazine (CMZ), a clathrin‐mediated endocytosis inhibitor; genistein (GEN), a caveolae‐mediated endocytosis inhibitor; amiloride (AMI), a macropinocytosis inhibitor; and methyl‐β‐cyclodextrin (Mβ‐CD), an inhibitor of lipid raft‐mediated endocytosis. Statistical significance was determined using one‐way ANOVA with Tukey's multiple comparisons test. Data are presented as mean ± SEM.

To explore whether some indirect pathways contribute to mRNA delivery to the brain, such as: (1) circulating immune cells, particularly blood and spleen macrophages, that could be transfected by LNPs and subsequently migrate to distal tissues [32, 33], (2) mRNA‐containing exosomes released from LNP‐transfected cells might reach the brain [34, 35, 36], and (3) LNPs might target transferrin receptor 1 (TfR1) which is highly expressed in brain endothelial cells, thereby participating in receptor‐mediated transcytosis [37], we conducted a series of depletion and blocking experiments. We first examined the roles of macrophages and exosomes in mediating LNP delivery to the brain. Mice were treated with clodronate liposomes to deplete macrophages [38] and with GW4869 [39] to inhibit exosome production, followed by intravenous administration of BBB‐targeting LNPs. Neither intervention significantly affected mRNA delivery to the brain, suggesting that these pathways are not essential for LNP transport (Figure 5f,g). We next investigated the involvement of TfR1 in BBB‐targeting. However, pretreatment with an anti‐TfR1 antibody had no measurable effect on delivery efficiency, indicating that TfR1‐mediated transcytosis is unlikely to be the dominant mechanism in this context in our case (Figure 5h). These results collectively suggest that BBB‐targeting LNPs do not depend on macrophage migration, exosome‐mediated transfer, or TfR1‐associated uptake.

Given these results, we decided to investigate whether surface‐bound serum proteins may contribute to the BBB‐targeting capabilities of these novel LNPs. When administered intravenously, LNPs bind plasma proteins to create an interfacial layer known as “protein corona”, which influences their biological identity and tissue tropism by modulating interactions with specific cells and organs [40, 41]. To investigate hypothesized endogenous targeting mechanism for BBB‐targeting LNPs, we analyzed the specific protein compositions adsorbed to LNPs using liquid chromatography‐tandem mass spectrometry (LC‐MS/MS) following an established protocol [42]. Results show that vitronectin, albumin, apolipoprotein A‐I, and fibrinogen were consistently enriched in the protein coronas of all evaluated LNPs (Figure 5i,j). Compared to C6O2 LNPs and Lung‐SORT LNPs, BBB‐targeting LNPs exhibited a significantly higher abundance of apolipoprotein A‐I (ApoA‐I) (Figure 5k). ApoA‐I is a key endogenous ligand known to participate in BBB transport, which has been reported to associate with cellular uptake via a lipid raft‐mediated pathway [13]. Its enrichment in the protein corona of BBB‐targeting LNPs suggests a potential role in facilitating their uptake. To examine this, we performed endocytic pathway inhibition experiments. Pre‐treatment with methyl‐β‐cyclodextrin (Mβ‐CD), which disrupts lipid rafts, partially suppressed mRNA delivery by BBB‐targeting LNPs, indicating partial reliance on lipid raft‐mediated uptake. In contrast, inhibition of macropinocytosis (amiloride), clathrin‐mediated endocytosis (chlorpromazine), or caveolae‐mediated endocytosis (genistein) had minimal effect on LNP internalization, suggesting these pathways were not primarily involved (Figure 5l). The observed dependence on lipid raft‐mediated uptake aligns with previous findings that ApoA‐I can engage scavenger receptors (e.g., SR‐B1) to promote cholesterol‐mediated endocytosis in a clathrin‐independent manner [43].

Together, these results suggest that the BBB‐targeting capability of the LNPs may be driven by a dual mechanism: enhanced endosomal escape enabled by rational lipid design, and endogenous targeting facilitated by ApoA‐I adsorption, which promotes lipid raft‐mediated uptake at the BBB. These findings highlight the importance of both lipid structural optimization and protein corona composition in directing LNP biodistribution, providing a mechanistic basis for designing materials and formulations with efficient and selective brain delivery.

## 3 Conclusion

In this study, we developed a ligand‐free LNP platform that enables efficient and selective delivery of mRNA to brain endothelial cells through noninvasive systemic administration. By integrating rational design of cholesterol‐conjugated amino lipids with a modular synthesis strategy, we generated a chemically diverse library of 51 BBB‐targeting lipids. Systematic in vitro and in vivo evaluation of 486 LNPs identified the optimized four‐component formulation—comprising BBB‐targeting lipid C6O2B2, DODAP, DSPC, and DMG‐PEG, without the addition of free cholesterol—that achieved high transfection efficiency across the brain vascular network, particularly the brain endothelium, without compromising the integrity and function of BBB. Mechanistic investigations revealed that the BBB‐targeting capability may be driven by a dual mechanism involving enhanced endosomal escape facilitated by the unique lipid structural features, and endogenous targeting mediated by ApoA‐I, a plasma protein enriched in the LNP's protein corona. Importantly, the platform avoids neuronal and glial transfection, offering a safer and more controlled approach for modulating endothelial function, reducing neuroinflammation, or restoring vascular homeostasis—without the risks associated with broader CNS exposure.

In many therapeutic contexts, particularly during early or chronic stages of neurological diseases, it may be neither necessary nor desirable to cross the BBB and expose the CNS to circulating agents. Instead, modulating the BBB itself can offer therapeutic benefit. However, achieving safe and efficient gene delivery specifically to brain endothelial cells remains challenging. Our work introduces a class of cholesterol‐based lipids that enhance both the structural stability of LNPs and their BBB‐targeting efficiency. The resulting LNP platform is simple, scalable, and achieves potent brain transfection without incorporating free cholesterol, which distinguishes it from conventional LNPs. This system enables effective cerebrovascular gene delivery that does not rely on receptor targeting or BBB disruption. By combining rational lipid design with mechanistic insight, we provide both new materials and valuable guidance for precision delivery of RNA therapeutics to the brain endothelium. This approach holds promise for a range of applications to be pursued in the future, including treatment of cerebrovascular disorders, delivery of neuroprotective factors, and modulation of neurovascular inflammation, laying the foundation for next‐generation BBB‐targeted RNA therapies.

Despite these promising results, several limitations should be acknowledged. Although preferential delivery to the brain was achieved, transfection was not exclusively restricted to the BBB, and some endothelial uptake was observed in peripheral tissues, such as the heart. In addition, while enhanced delivery to brain endothelial cells was demonstrated, the underlying mechanisms require deeper understanding. Future studies will focus on further improving targeting specificity and refining LNP design, elucidating the mechanistic basis of BBB selectivity, and validating therapeutic efficacy across a broader range of diverse disease models.

## 4 Experimental Section

### 4.1 Materials

All chemicals and reagents were of analytical grade and used without further purification. Amines, cholesterol, Evans blue, Dulbecco's modified phosphate buffered saline (PBS), RPMI‐1640 medium, fetal bovine serum (FBS), and trypsin‐EDTA (0.25%) were obtained from Sigma‐Aldrich. Lipids, including 1,2‐dioleoyl‐sn‐glycero‐3‐phosphoethanolamine (DOPE), 1,2‐dioleoyl‐sn‐glycero‐3‐phosphoglycerol (DOPG), sphingomyelin (Egg SM), 1,2‐distearoyl‐sn‐glycero‐3‐phosphocholine (DSPC), 1,2‐dioleoyl‐sn‐glycero‐3‐phosphocholine (DOPC), and 1,2‐dimyristoyl‐sn‐glycero‐3‐phosphocholine (DMPC) were sourced from Avanti Polar Lipids. 1,2‐dimyristoyl‐rac‐glycero‐3‐methoxy(poly(ethylene glycol))‐2000 (DMG‐PEG2000) was purchased from NOF America Corporation. The Quant‐iT RiboGreen RNA assay kit was obtained from Life Technologies. d‐Luciferin firefly, sodium salt monohydrate was acquired from Gold Biotechnology.

### 4.2 Synthesis and Purification of Synthesized Cores

Synthesized cores were synthesized through conjugation of amine headgroups with hydrophobic tails via acrylate chemistry. Specifically, each primary amine was designed to react with two equivalents of acrylate, while each secondary amine reacted with one equivalent. In brief, the amines were mixed with 2.4 or 1.2 equivalents of acrylate, respectively, and heated neat at 95°C for 24 h under stirring. Upon completion, the reaction mixture was cooled to room temperature and purified by column flash chromatography using a hexane/ethyl acetate (10:1) solvent system.

### 4.3 Synthesis and Purification of BBB‐Targeting Lipids

BBB‐targeting lipids were synthesized via a combinatorial approach, coupling commercially available cores or synthesized cores with cholesterol tails. In brief, cholesterol tails (1.0 equiv) and commercially available cores or synthesized cores (1.0 equiv) were dissolved in anhydrous acetonitrile in a sealed vial equipped with a stir bar. The reaction mixture was heated at 95°C for 24 h, followed by cooling to room temperature. The solvent was removed under reduced pressure, and the crude product was purified by column flash chromatography using a dichloromethane (DCM)/methanol (MeOH) (10:1) solvent system.

### 4.4 RNA Synthesis

Firefly luciferase mRNA and Cre mRNA were purchased from Hongene. IL‐10 mRNA was synthesized via In Vitro Transcription (IVT) with optimized protocols to ensure high yield and integrity. Linearized plasmids encoding the IL‐10 protein were prepared by PCR amplification and inserted into pCS2+MT vectors containing enhanced 5' and 3' untranslated regions (UTRs) and poly A sequences. IVT was performed using the MEGAscript SP6 transcription kit (Thermo Fisher), with N1‐methylpseudouridine‐5'‐triphosphate substituted for uridine triphosphate to produce mRNA with modified nucleosides, enhancing stability and reducing immunogenicity. The transcribed mRNA was subsequently capped (Cap1) using the Capping System from Hongene.

### 4.5 BBB‐Targeting LNP Formulation and Characterization

BBB‐targeting LNPs were prepared following a method similar to previous protocols [25]. In brief, mRNA was dissolved in a citrate buffer (10 mM, pH 4.5) to create the aqueous phase. The lipid components, including the BBB‐targeting lipid, helper lipids, DMG‐PEG2000, and DODAP, were dissolved in ethanol to form the organic phase. The two phases were rapidly combined at a 1:3 volumetric ratio under continuous vortex mixing and incubated at room temperature for 15 min to ensure uniform nanoparticle formation. The final lipid‐to‐mRNA weight ratio was maintained at 40:1. The resulting mRNA‐loaded LNPs were dialyzed against PBS (pH 7.4) using Pur‐A‐Lyzer midi dialysis chambers (Sigma‐Aldrich) for 1 h to remove residual organic solvent. Particle size, zeta potential, and polydispersity index (PDI) were determined by dynamic light scattering (Malvern MicroV model; He–Ne laser, wavelength = 632 nm). Encapsulation efficiency of mRNA was quantified using the Quant‐iT RiboGreen RNA assay kit (Invitrogen).

### 4.6 Isolation of Plasma Proteins Associated With LNPs

The protein corona was isolated using a method previously described by our group [41]. Briefly, BBB‐targeting LNPs were incubated with plasma from C57BL/6 mice at a 1:1 volume ratio, achieving a final lipid concentration of 1 mg/mL. The mixture was maintained at 37°C for 15 min to allow protein adsorption, then carefully layered onto a 0.7 M sucrose cushion and centrifuged at 15 000 g for 1 h at 4°C. The resulting pellet, containing LNP‐protein complexes, was washed 3 times with PBS to remove unbound proteins and resuspended in 2% SDS for protein solubilization. Excess lipids were efficiently removed using the ReadyPrep 2‐D Cleanup kit (Bio‐Rad). The purified proteins were stored at −80°C for subsequent proteomic analysis.

### 4.7 Lipid Fusion by FRET Assay

Endosomal‐mimicking anionic liposomes were prepared by dissolving a mixture of DOPS, DOPC, DOPE, NBD‐PE, and Rho‐PE (molar ratio 25:25:48:1:1) in chloroform, followed by vacuum drying to form a thin lipid film. The film was then resuspended in PBS (pH 7.4) to create liposomes, which were sonicated for 20 min (10‐s sonication and 10‐s rest cycles) to achieve a final lipid concentration of 1 mM. LNPs were prepared using the procedure described above. For the FRET assay, 100 µL of PBS (pH 5.5) was added to each well of a black‐bottom 96‐well plate, followed by the addition of 1 µL of the endosomal‐mimicking anionic liposomes. BBB‐targeting LNPs were added at a LNP‐to‐liposome ratio of 10:1. After incubating at 37°C, fluorescence measurements were taken using a microplate reader with excitation/emission wavelengths of 465/520 nm. Liposomes incubated with PBS and Triton X‐100 served as the negative control (*F*_min_) and positive control (*F*_max_), respectively. The lipid fusion percentage was calculated using the formula:

$$Lipid\;fusion\left(\%\right)=\left(F-F_{\min}\right)/\left(F_{\max}-F_{\min}\right)\times 100\%$$

### 4.8 In Vitro Delivery of Luc mRNA

BBB‐targeting LNPs were prepared using the pipette mixing method as previously described [25]. Luc mRNA LNP formulations were diluted to a concentration of 1 ng/µL mRNA and used for delivery to IGROV‐1 ovarian cancer cells, a cell line known to exhibit moderate resistance to transfection. IGROV‐1 cells were treated with BBB‐targeting LNP formulations containing Luc mRNA (15 ng mRNA per well, *n* = 5 per group) for 24 h. Cell viability and luciferase gene expression were assessed using the ONE‐Glo + Tox luciferase reporter and cell viability assay.

### 4.9 Preparation of Plasma Protein Samples for Mass Spectrometry

A 10 µL aliquot of plasma proteins was loaded onto a 12% Mini‐PROTEAN TGX 25 Precast Protein Gel (Bio‐Rad) and subjected to electrophoresis at 90 V for 10 min. The gel was stained with Bio‐Safe Coomassie Stain (Bio‐Rad) for 1 h and destained in deionized water for 1 h. Distinct protein bands were excised, diced into approximately 1 mm^3^ pieces, and submitted to the Proteomics Core at the University of Texas Southwestern for mass spectrometry analysis. Proteins were identified and ranked based on their relative abundance and molecular weight (Mw). A custom Python script was employed to generate a heat map highlighting the most abundant proteins.

### 4.10 Animal Experiments

All animal procedures were approved by the Institutional Animal Care and Use Committee (IACUC) at the University of Texas Southwestern Medical Center and conducted in accordance with local, state, and federal regulations. C57BL/6 mice were obtained from Charles River Laboratories. B6.Cg‐Gt(ROSA)26Sortm14(CAG‐tdTomato)Hze/J mice, commonly referred to as Ai14 or Ai14(RCL‐tdT) mice, were sourced from The Jackson Laboratory (Catalog No. 007914) and bred for homozygous expression of the Cre reporter allele.

### 4.11 In Vivo Delivery of Luciferase mRNA

Six‐to‐eight‐week‐old C57BL/6 mice (18–20 g) were intravenously administered 0.1 mg/kg of Luciferase (Luc) mRNA encapsulated in lipid nanoparticles (LNPs). Six hours post‐injection, the animals were given an intraperitoneal injection of D‐Luciferin (150 mg/kg) and anesthetized with 2.5% isoflurane in oxygen. Imaging was performed using the AMI‐HTX system (Spectral Instruments Imaging). Following live animal imaging, the primary organs were harvested for ex vivo imaging.

### 4.12 In Vivo Delivery of Cre mRNA

Six‐to‐eight‐week‐old Ai14 mice (18–20 g) received intravenous injections of Cre mRNA LNPs at doses of 0.3 mg/kg on Days 0 and 2. Forty‐eight hours after the final injection, the mice were euthanized, and their organs were imaged using the AMI‐HTX system (Spectral Instruments Imaging).

### 4.13 MR Image Acquisition

MR imaging was performed on a 7T small animal MRI scanner (Bruker BioSpin GmbH) using a 35 mm quadrature transmit receiver coil. Mice were anesthetized with 1.5% isoflurane. A close warm‐water circuit maintained the body temperature at 37°C. Gadobutrol (Gadovist, Bayer Healthcare Pharmaceuticals) was administered via tail catheter (1.0 mmol/kg) followed by a 70 µL saline flush. T1 quantification was done using a variable repetition time (TR) RARE sequence. Effective TE = 7 ms, TR = 5500, 3000, 1500, 800, 400, 205 ms, slice thickness = 0.8 mm, in‐plane resolution = 0.1 mm x 0.2 mm, RARE factor = 2, number of averages = 1. Post‐Gd T1 images were acquired 10 min after the administration of Gd. T1 maps were constructed on the scanner using a vendor provided software.

### 4.14 Immunofluorescence

Mice were perfused with 4% paraformaldehyde (PFA), and tissues were post‐fixed in 4% PFA at 4°C overnight. Tissues were then cryoprotected by immersion in 30% sucrose for 48 h, followed by embedding in optimal cutting temperature (OCT) compound. Cryosections (20 µm) were prepared using a Leica cryostat (CM3050S). Sections were fixed in cold PFA for 20 min and subsequently rinsed in PBS 3 times. Blocking was performed with 5% bovine serum albumin (BSA) and 0.1% Triton X‐100 in PBS for 1 h at room temperature. Primary antibodies—Anti‐NeuN (Rabbit mAb #ab177487, abcam), Anti‐IBA1 (Rabbit mAb #019‐19741, FUJIFILM Wako Pure Chemical Corporation), and Anti‐CD31 (Rabbit mAb #PA5‐32321, Thermo Fisher)—were applied at the appropriate concentrations in staining buffer (1% BSA/0.1% Triton X‐100 in PBS) and incubated overnight at 4°C. After washing, sections were incubated with secondary antibodies (Alexa Fluor–conjugated secondary antibodies were from Invitrogen) for 1 h at room temperature. Slides were mounted with ProLong Gold Mountant with DAPI to visualize cell nuclei. Imaging was performed using confocal microscopy (Zeiss LSM 700), and images were analyzed with ZEN 2010 software (version 6.0.62, Carl Zeiss MicroImaging).

### 4.15 In Vivo IL‐10 mRNA Delivery

C57BL/6 mice received intravenous injections of PBS or C6O2B2 LNPs encapsulating IL‐10 mRNA (0.5 mg/kg) at 24 and 3 h prior to LPS challenge. Acute brain injury was induced by intraperitoneal administration of LPS derived from Escherichia coli (O111:B4, Sigma) at a dose of 1.2 mg per mouse (defined as 0 h). Six hours after LPS injection, 2% Evans Blue dye was administered via retro‐orbital injection. After 2 h of circulation, mice were deeply anesthetized and perfused transcardially with PBS. Brains were then harvested and imaged.

### 4.16 qPCR

Total RNA was extracted from approximately 20–30 mg of lung tissue using 800 µL of ice‐cold TRIzol reagent (Invitrogen, 15596026). Samples were incubated at room temperature for 5–10 min with occasional vortexing to ensure complete lysis. Chloroform (160 µL) was then added, followed by vigorous mixing and incubation at room temperature for 2 min. The lysates were centrifuged at 12 000 × g for 15 min at 4°C to separate phases. The upper aqueous layer was carefully collected and combined with an equal volume of 70% ethanol. RNA purification was carried out using the RNeasy Mini Kit (Qiagen, 74104) according to the manufacturer's instructions. Purified RNA was eluted in 30–50 µL of nuclease‐free water, and concentrations were measured using a NanoDrop 1000 spectrophotometer (Thermo Fisher Scientific). For cDNA synthesis, 1 µg of total RNA was reverse transcribed using iScript Reverse Transcription Supermix (Bio‐Rad, 1708841) on a SimpliAmp Thermal Cycler (Thermo Scientific, A24811). Quantitative PCR was performed on a QuantStudio 5 Real‐Time PCR System (Applied Biosystems, A28140) using iTaq Universal SYBR Green Supermix (Bio‐Rad, 1725121). Primer sequences are provided in Table S2. Specificity and amplification efficiency were confirmed by melt curve analysis. Gene expression levels were normalized to actin and calculated using the 2^−ΔΔCt^ method.

### 4.17 Coding Sequences for Mouse IL‐10

Atgcctggctcagcactgctatgctgcctgctcttactgactggcatgagga tcagcaggggccagtacagccgggaagacaataactgcacccacttcccagtcggccagagccacatgctcctagagctgcggactgccttcagccaggtgaagactttctttcaaacaaaggaccagctggacaacatactgctaaccgactccttaatgcaggactttaagggttacttgggttgccaagccttatcggaaatgatccagttttacctggtagaagtgatgccccaggcagagaagcatggcccagaaatcaaggagcatttgaattccctgggtgagaagctgaagaccctcaggatgcggctgaggcgctgtcatcgatttctcccctgtgaaaataagagcaaggcagtggagcaggtgaagagtgattttaataagctccaagaccaaggtgtctacaaggccatgaatgaatttgacatcttcatcaactgcatagaagcatacatgatgatcaaaatgaaaagctaa.

### 4.18 Statistical Analysis

Statistical analyses were conducted using GraphPad Prism v.10 (GraphPad Software). Data are expressed as mean ± SEM. One‐way ANOVA followed by Tukey's multiple comparisons test was used for statistical analysis. A p‐value of <0.05 was considered statistically significant.

## Author Contributions

Z.T., X.W., and D.J.S. designed the research. Z.T., X.W., A.P.B., E.G., J.W., and L.F. performed the experiments. D.J.S. supervised the experiments and managed the project. Z.T., X.W., L.F., and D.J.S. wrote the manuscript. All the authors were involved in the data analyses and the improvement of the manuscript.

## Conflicts of Interest

Daniel J. Siegwart discloses conflicts of interests in ReCode Therapeutics, Signify Bio, Jumble Therapeutics, and Pegasus Bio. Lukas Farbiak discloses conflicts of interest in Signify Bio. The remaining authors declare no conflicts of interest.

## Supporting information




**Supporting File**: adma74762‐sup‐0001‐SuppMat.pdf.

## Data Availability

The data that support the findings of this study are available from the corresponding author upon reasonable request.
