# Supplementary material for: Esterified Cholesterol Conjugates Enable LNP‐Mediated mRNA Delivery to the Blood–Brain Barrier Following Intravenous Administration
Source: Adv Mater. 2026 Aug 23;38(59):e74762. doi: 10.1002/adma.74762 (PMC13689884; doi:10.1002/adma.74762)
Supplement: Supplementary file 1 [file ADMA-38-e74762-s001.pdf]

Supporting Information

Esterified cholesterol conjugates enable LNP-mediated mRNA delivery to the blood-brain barrier following intravenous administration

Zeru Tian[1,2], Xu Wang[1,2], Alexis Pacheco Benitez[1], Erick Guerrero[1], Yaou Duan[1], Jingwen Wei[1], Lukas Farbiak[1], Daniel J. Siegwart[1,*]

[1]Department of Biomedical Engineering, Department of Biochemistry, Simmons Comprehensive Cancer Center, Program in Genetic Drug Engineering, University of Texas Southwestern Medical Center, Dallas, TX, USA. [2]These authors contributed equally: Zeru Tian, Xu Wang.

*E-mail: Daniel.Siegwart@UTSouthwestern.edu

The Supplementary information includes:

Figures S1-S18

Tables S1-S2

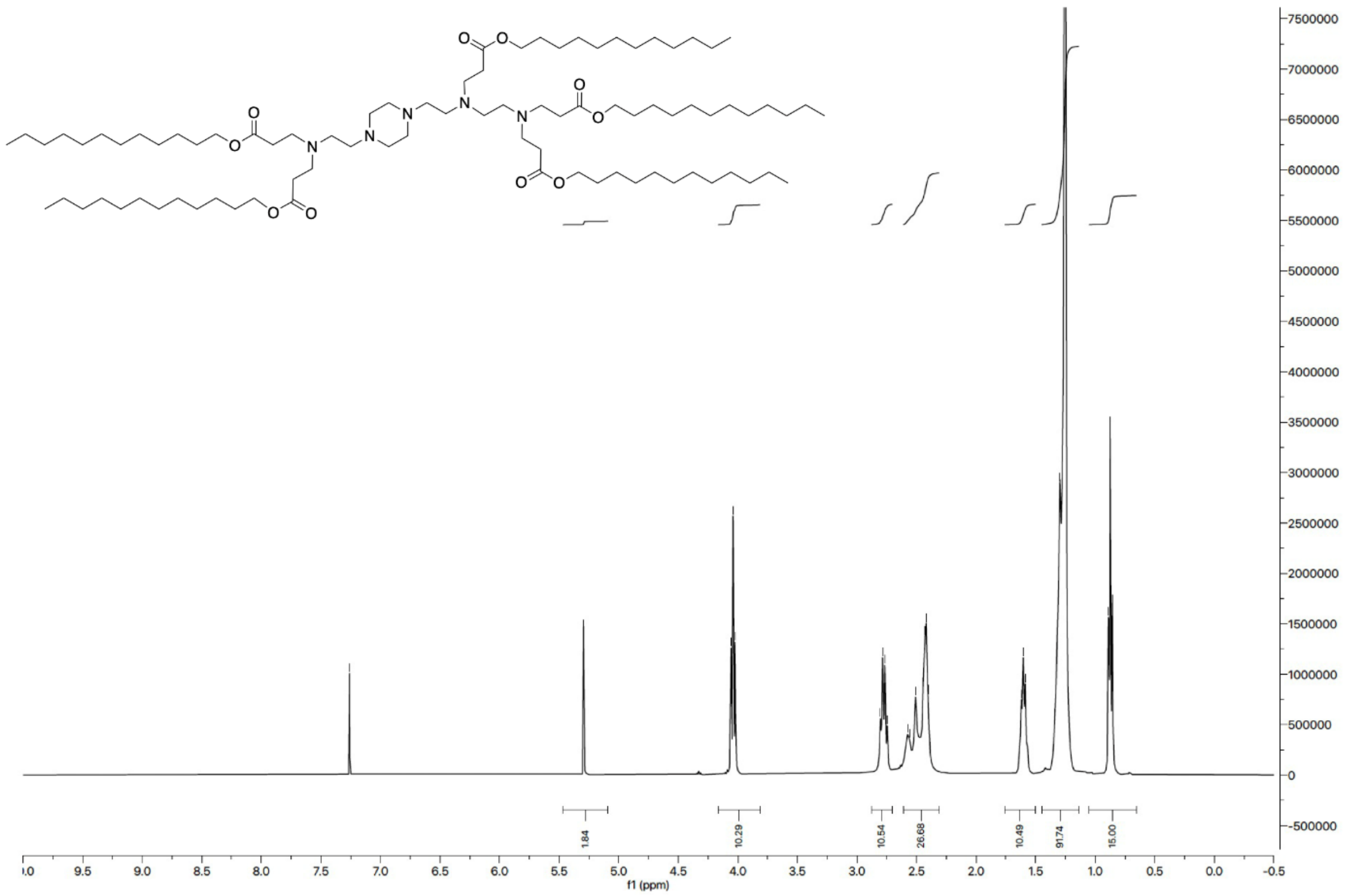

Figure S1. ^1H NMR spectra of C6O2 in $CDCl_3$. ^1H NMR (400 MHz, $CDCl_3$) δ 5.29 (d, *J* = 1.2 Hz, 2H), 4.04 (t, *J* = 6.8 Hz, 10H), 2.77 (q, *J* = 7.7 Hz, 10H), 2.61 – 2.31 (m, 26H), 1.60 (t, *J* = 7.0 Hz, 10H), 1.27 (m, 92H), 0.87 (t, *J* = 6.7 Hz, 15H).

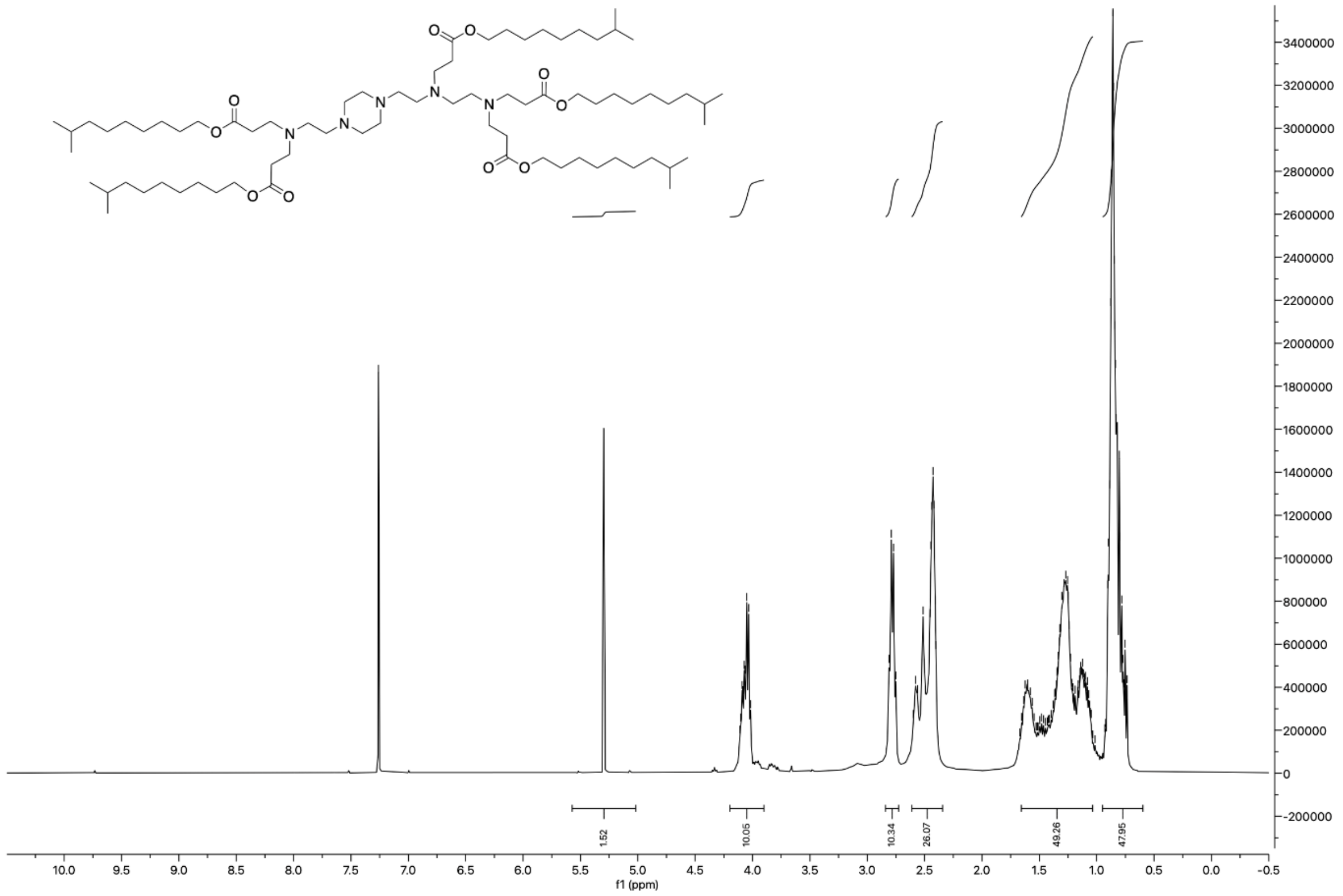

Figure S2. ^{1}H NMR spectra of C6O1 in $CDCl_3$. ^{1}H NMR (400 MHz, $CDCl_3$) δ 5.30 (s, 2H), 4.11 – 4.02 (m, 10H), 2.78 (q, *J* = 7.7 Hz, 10H), 2.61 – 2.34 (m, 26H), 1.66 – 1.03 (m, 49H), 0.95 – 0.60 (m, 48H).

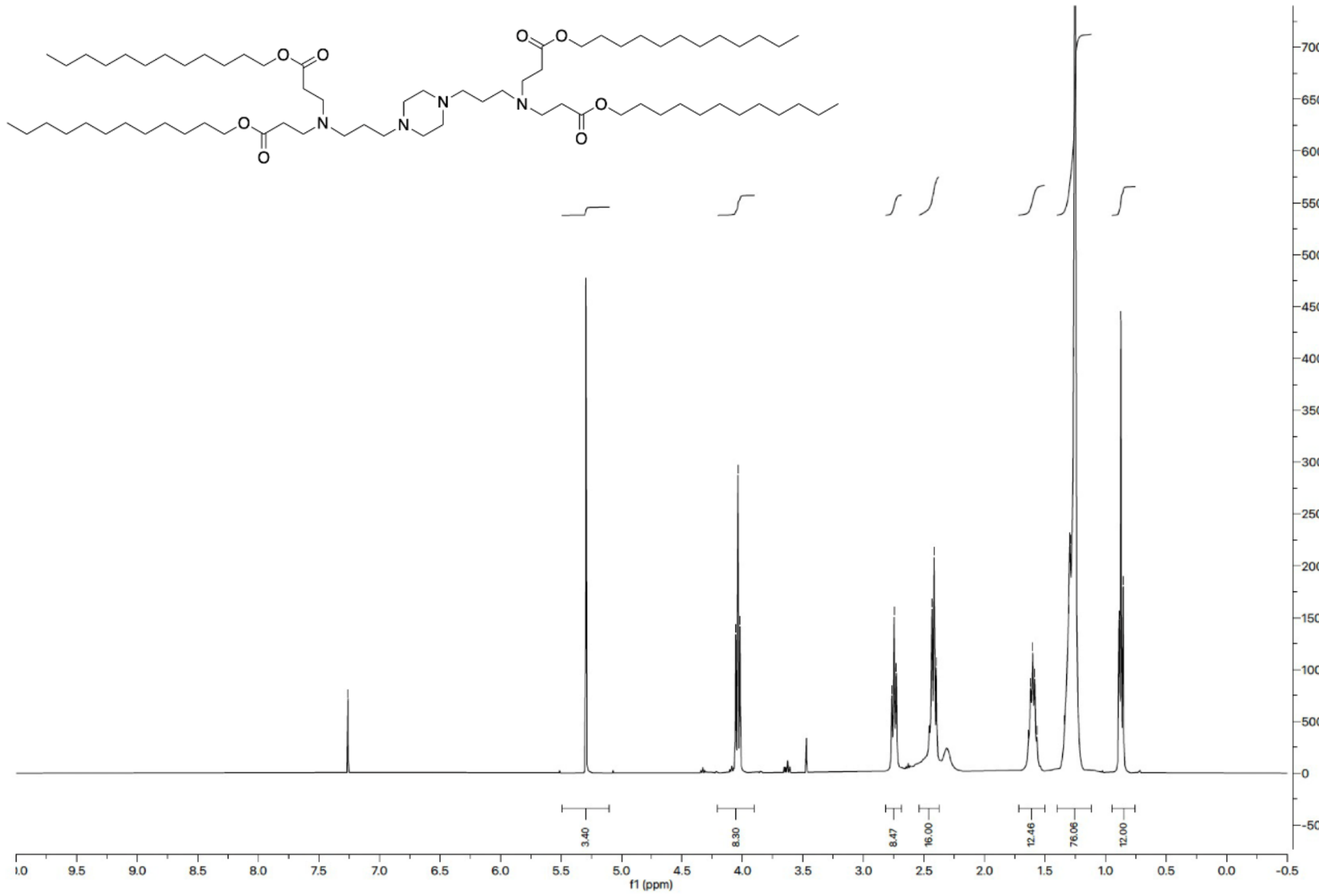

Figure S3. ^{1}H NMR spectra of C5O2 in $CDCl_3$. ^{1}H NMR (400 MHz, $CDCl_3$) δ 5.29 (s, 4H), 4.04 (t, *J* = 6.8 Hz, 8H), 2.75 (t, *J* = 7.2 Hz, 8H), 2.54 – 2.38 (m, 16H), 1.64–1.57 (m, 12H), 1.40 – 1.12 (m, 76H), 0.95 – 0.75 (m, 12H).

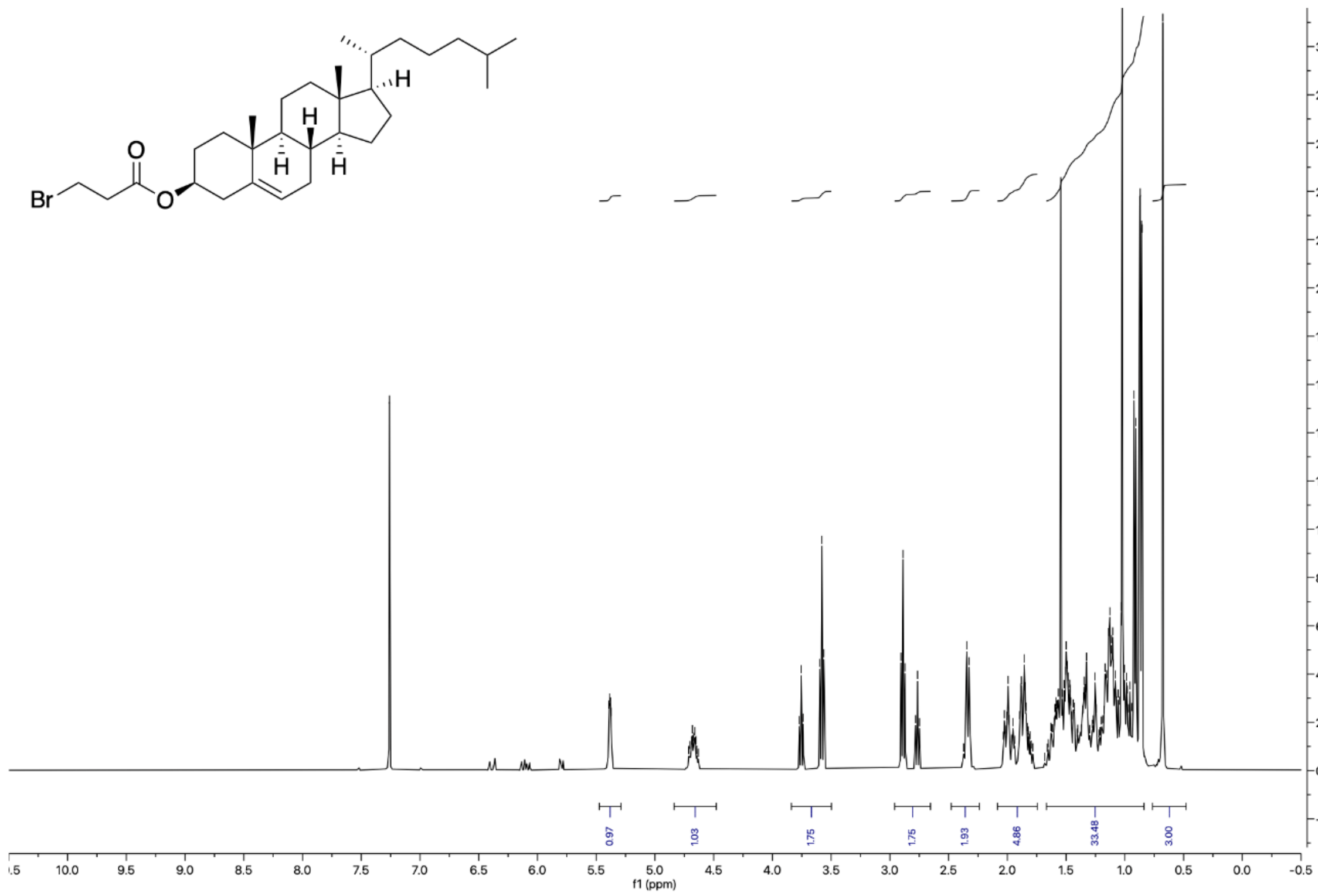

Figure S4. ^{1}H NMR spectra of B1 in $CDCl_3$. ^{1}H NMR (400 MHz, $CDCl_3$) δ 5.38 (dd, J = 5.3, 2.0 Hz, 1H), 4.71 – 4.63 (m, 1H), 3.58 (t, J = 6.9 Hz, 2H), 2.89 (t, J = 6.9 Hz, 2H), 2.34 (d, J = 8.1 Hz, 2H), 2.08 – 1.75 (m, 5H), 1.67 – 0.84 (m, 33H), 0.68 (s, 3H).

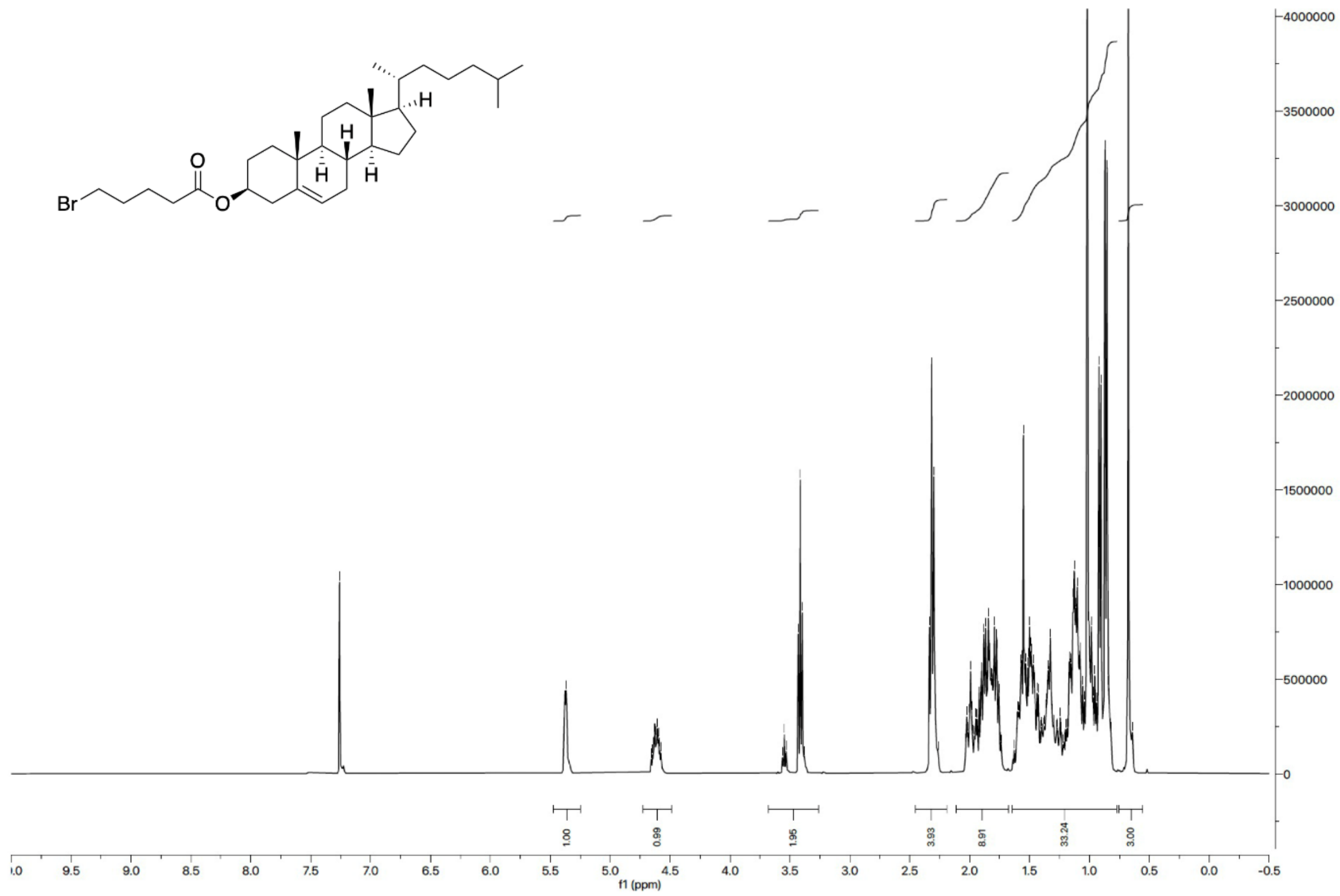

Figure S5. ^{1}H NMR spectra of B2 in $CDCl_3$. ^{1}H NMR (400 MHz, $CDCl_3$) δ 5.47 – 5.25 (m, 1H), 4.66 - 4.58 (m, 1H), 3.68 – 3.26 (m, 2H), 2.32 (t, J = 7.2 Hz, 4H), 2.11 – 1.67 (m, 9H), 1.65 – 0.77 (m, 33H), 0.68 (s, 3H).

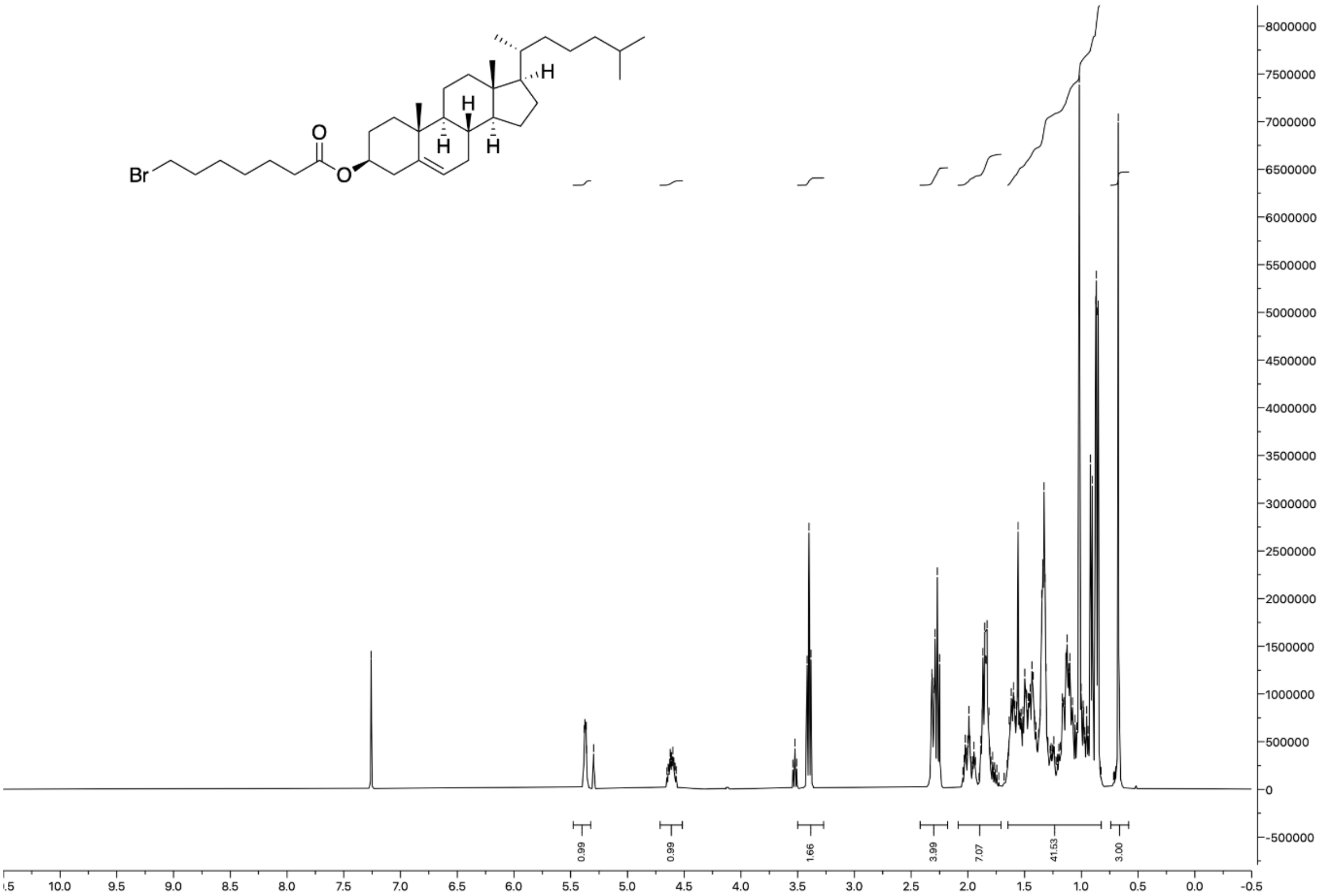

Figure S6. ^{1}H NMR spectra of B3 in $CDCl_3$. ^{1}H NMR (400 MHz, $CDCl_3$) δ 5.48 – 5.24 (m, 1H), 4.61 (m, 1H), 3.46 (m, 2H), 2.42 – 2.18 (m, 4H), 2.08 – 1.71 (m, 7H), 1.65 – 0.82 (m, 41H), 0.67 (s, 3H).

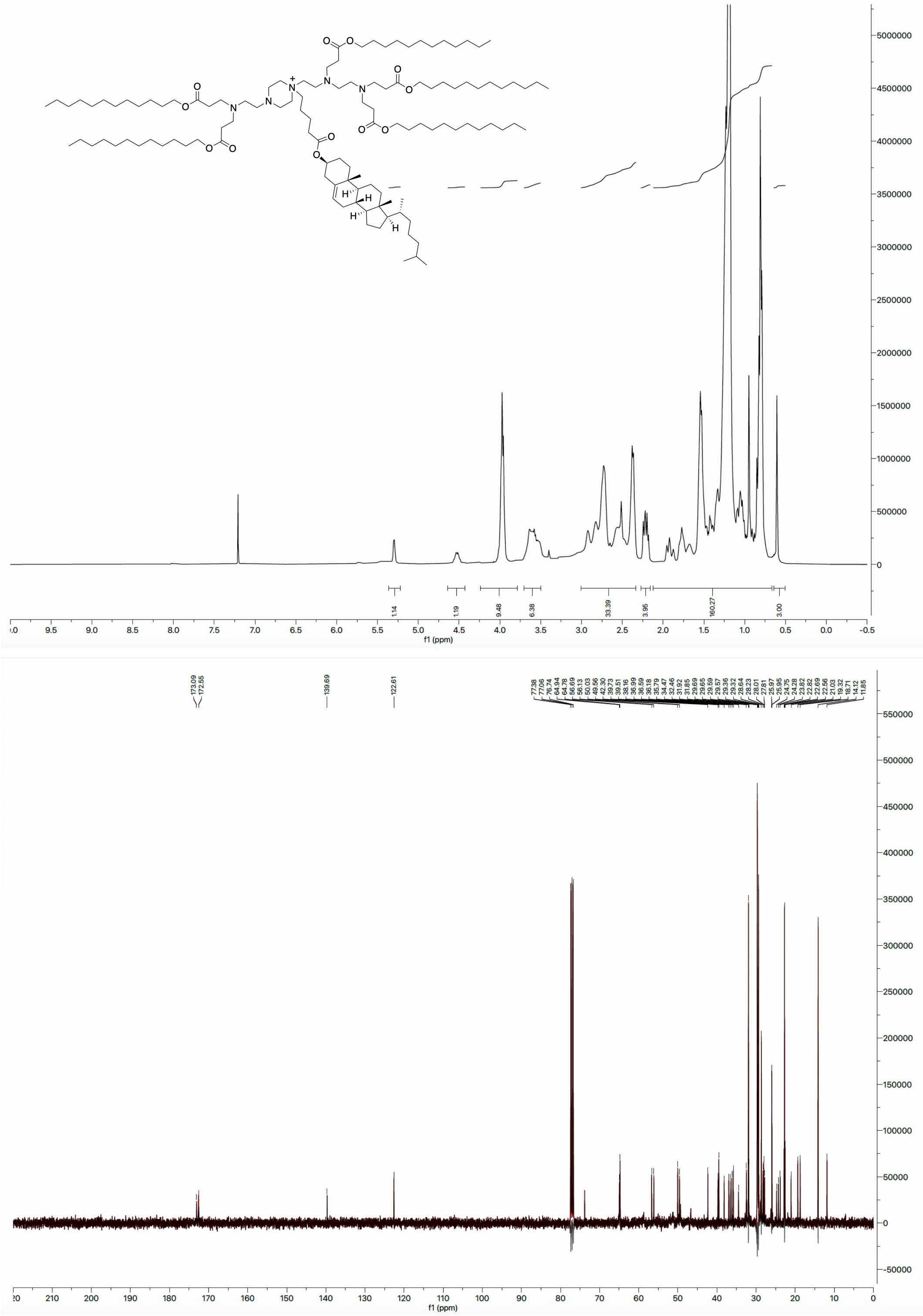

Figure S7. ^{1}H NMR and ^{13}C NMR spectra of C6O2B2 in $CDCl_3$. ^{1}H NMR (400 MHz, $CDCl_3$) δ 5.30 (d, *J* = 5.0 Hz, 1H), 4.54 – 4.51 (m, 1H), 3.99 – 3.95 (m, 10H), 3.64 – 3.51 (m, 6H), 2.94 – 2.35 (m, 33H), 2.24 – 2.18 (m, 4H), 1.96 – 0.78 (m, 160H), 0.61 (s, 3H), 0.87 (t, *J* = 6.7 Hz, 15H). ^{13}C NMR (101 MHz, $CDCl_3$) δ 173.09, 172.55, 139.69, 122.61, 64.94, 64.78, 56.69, 56.13, 50.03, 49.56, 42.30, 39.73, 39.51, 38.16, 36.99, 36.59, 36.18, 35.79, 34.47, 32.46, 31.92, 31.85, 29.69, 29.65, 29.59, 29.57, 29.36, 29.32, 28.64, 28.23, 28.01, 27.81, 25.97, 25.95, 24.75, 24.28, 23.82, 22.82, 22.69, 22.56, 21.03, 19.32, 18.71, 14.12, 11.85.

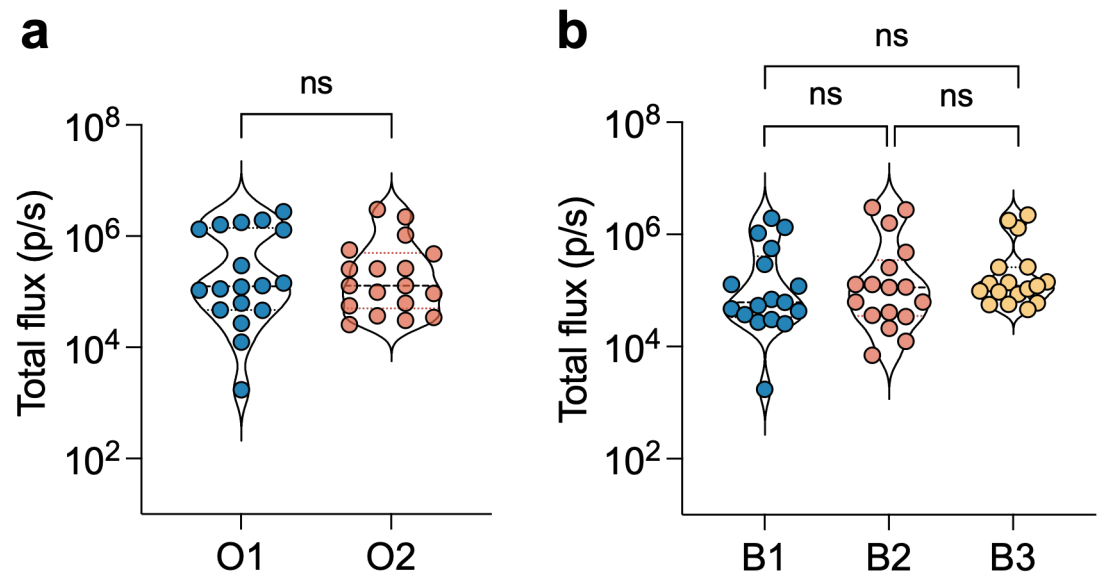

Figure S8. (**a**) Comparison of luciferase expression levels in LNPs formulated with O1 or O2 alkyls. (**b**) Effect of B1, B2, or B3 tails on luciferase activity. Statistics in (**a**,**b**), Student's *t*-test. Data are presented as mean ± s.e.m. ns: not significant.

Structure-function relationship analysis

In addition to luciferase expression, we included a hit rate analysis, defined as the fraction of LNP formulations achieving a brain total flux greater than 1×10^6 p/s. This analysis revealed a higher proportion of effective formulations among piperazine-containing lipids compared to those lacking the piperazine motif (**Figure S9a**), supporting the importance of the piperazine structure. We then compared two piperazine cores (C5 and C6) and found that the C6 core consistently outperformed C5, exhibiting both higher brain expression (**Figure S9b**) and an increased hit rate (**Figure S9c**). Together, these results identify the piperazine core, particularly the C6 structure, as a key determinant of delivery efficiency.

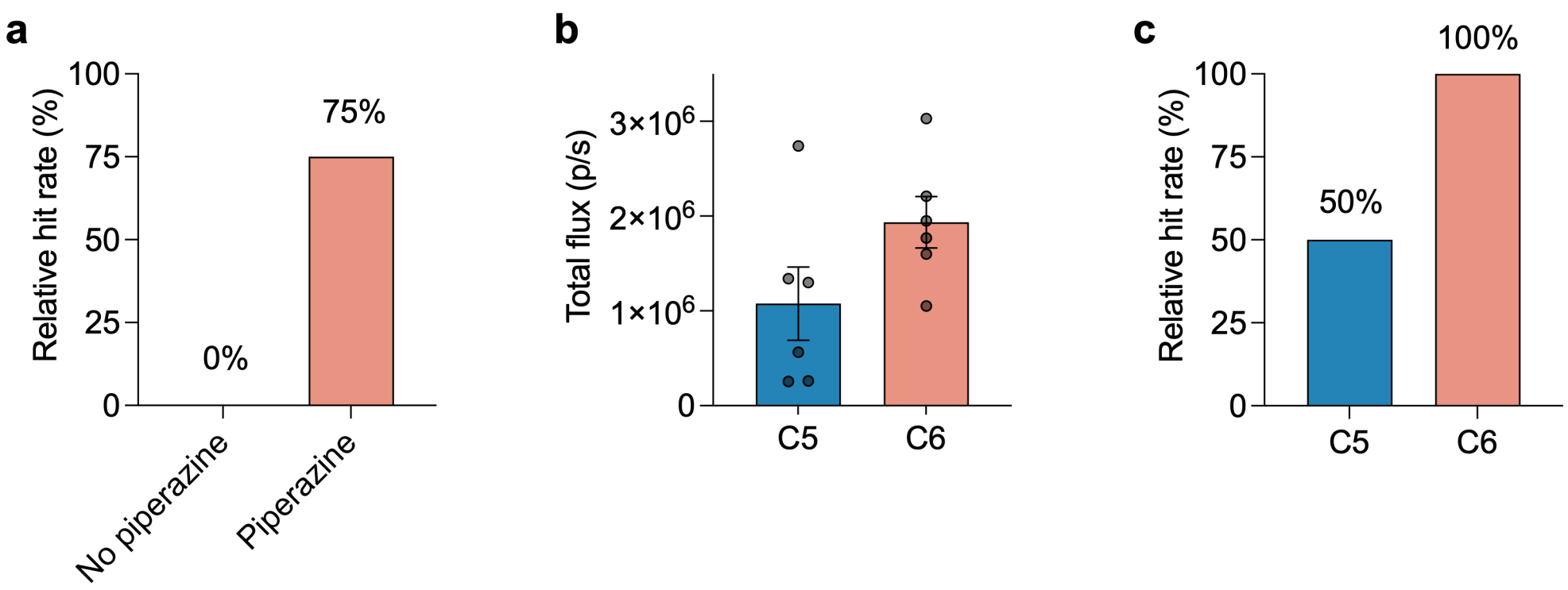

Figure S9. Structure-function relationship analysis of piperazine-containing lipids. (**a**) Hit rate analysis shows a higher proportion of effective formulations among piperazine-containing lipids compared to those lacking the piperazine motif. (**b**) LNPs formulated with the piperazine core C6 exhibit higher luciferase expression levels than those with the C5 core. (**c**) Hit rate analysis further demonstrates that incorporation of the C6 core increases the frequency of effective BBB-targeting delivery. LNP formulations achieving a brain total flux greater than 1×10^6 p/s were defined as hits for hit rate calculation.

Membrane fusion and endosomal escape

To evaluate how variations in the piperazine core, cholesterol-derived tails, and alkyl chains influence endosomal escape, we conducted a fluorescence resonance energy transfer (FRET) assay to monitor membrane fusion. Endosome mimicking liposomes were co-labelled with 1,2-dioleoyl-sn-glycero-3-phosphoethanolamine-N-(7-nitro-2-1,3-benzoxadiazol-4-yl) (NBD-PE, donor fluorophore) and 1,2-dioleoyl-sn-glycero-3-phosphoethanolamine-N-(lissamine rhodamine B sulfonyl) (Rho-PE, acceptor fluorophore), where close proximity between the FRET pair resulted in quenched NBD fluorescence. Upon membrane fusion, increased donor–acceptor distance led to recovery of the NBD signal.

We compared LNPs containing piperazine-based lipids in variations in alkyl chains (O1 vs. O2), cholesterol-derived tails (B1, B2, B3), and piperazine cores (C5 vs. C6). Quantitative analysis at 240 min of all these LNPs are shown in **Figure S10a**. Substitution with O1 or O2 alkyl chains and variation in tail structures (B1, B2, and B3) had minimal impact on membrane fusion (**Figure S10b,c**). In contrast, the piperazine core played a dominant role, with the C6 core exhibiting significantly enhanced membrane fusion compared to C5 (**Figure S10d**). Overall, C6O2B2 LNPs consistently showed the highest membrane fusion capability (**Figure S10a**).

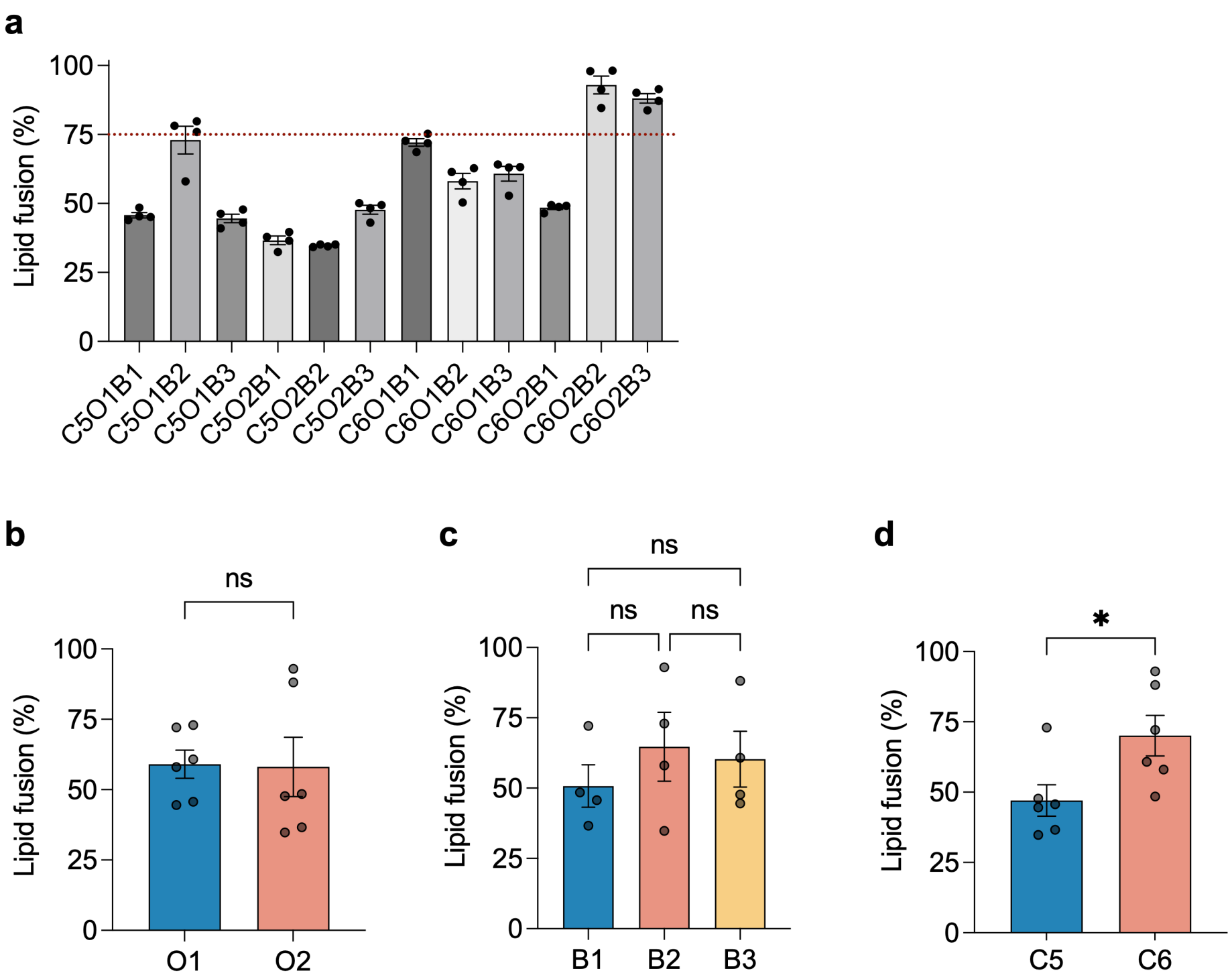

Figure S10. Membrane fusion and endosomal escape analysis of piperazine-containing LNPs. (**a**) Membrane fusion of LNPs containing piperazine-based lipids were determined by a FRET assay at pH 5.5 after 240 min. (**b**) Summary of membrane fusion for LNPs with different alkyl chains (O1 vs. O2). (**c**) Summary of membrane fusion for LNPs with different cholesterol-derived tails (B1-B3). (**d**) Summary of membrane fusion for LNPs containing different piperazine cores (C5 vs. C6). Statistics in (**b**,**c**), one-way ANOVA and Tukey's multiple comparisons test. Statistics in (**d**), Student's *t*-test. Data are presented as mean ± s.e.m. $^{*}P <$ 0.1, ns: not significant.

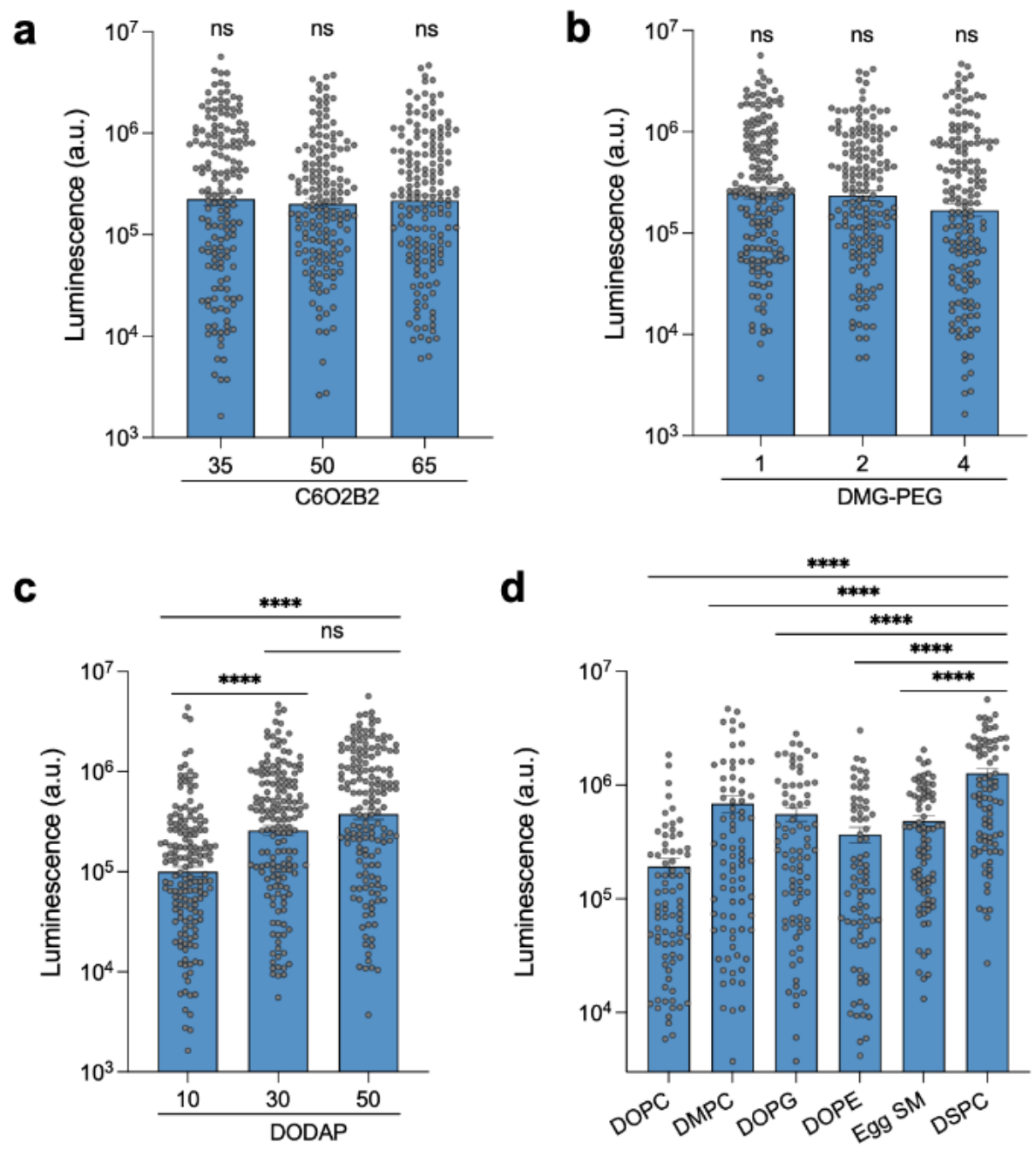

Figure S11. Luciferase activity of LNPs formulated with varying molar ratios of (**a**) C6O2B2, (**b**) DMG-PEG, and (**c**) DODAP, as well as (**d**) different helper lipids.

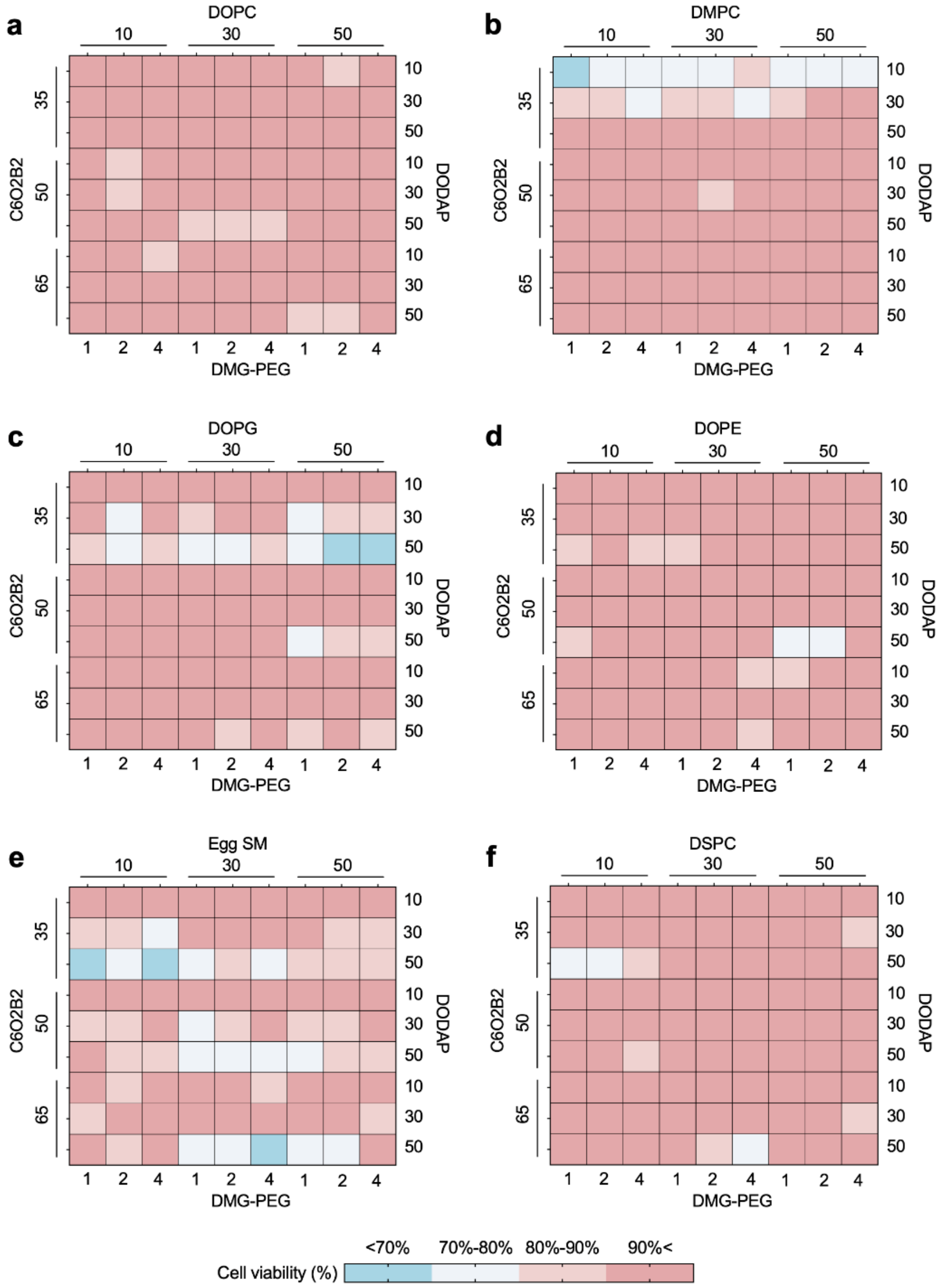

Figure S12. Cell viability of IGROV1 cells following treatment with Luc mRNA-containing brain LNPs formulated with different helper lipids: (**a**) DOPC, (**b**) DMPC, (**c**) DOPG, (**d**) DOPE, (**e**) EggSM, and (**f**) DSPC for 24 h. The majority of brain-targeting LNPs exhibited minimal cytotoxicity, with cell viability exceeding 80%.

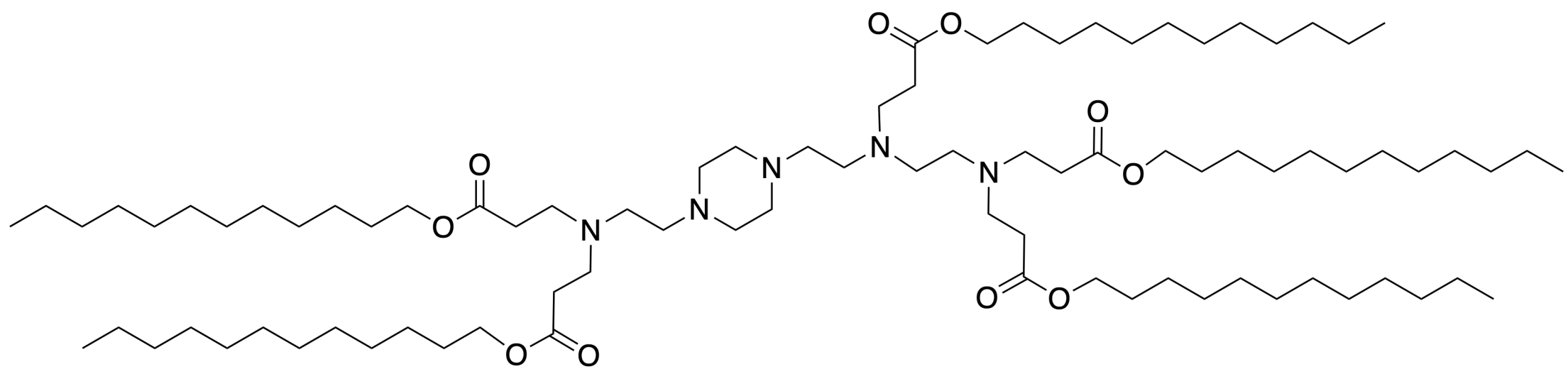

Figure S13. Chemical structure of C6O2.

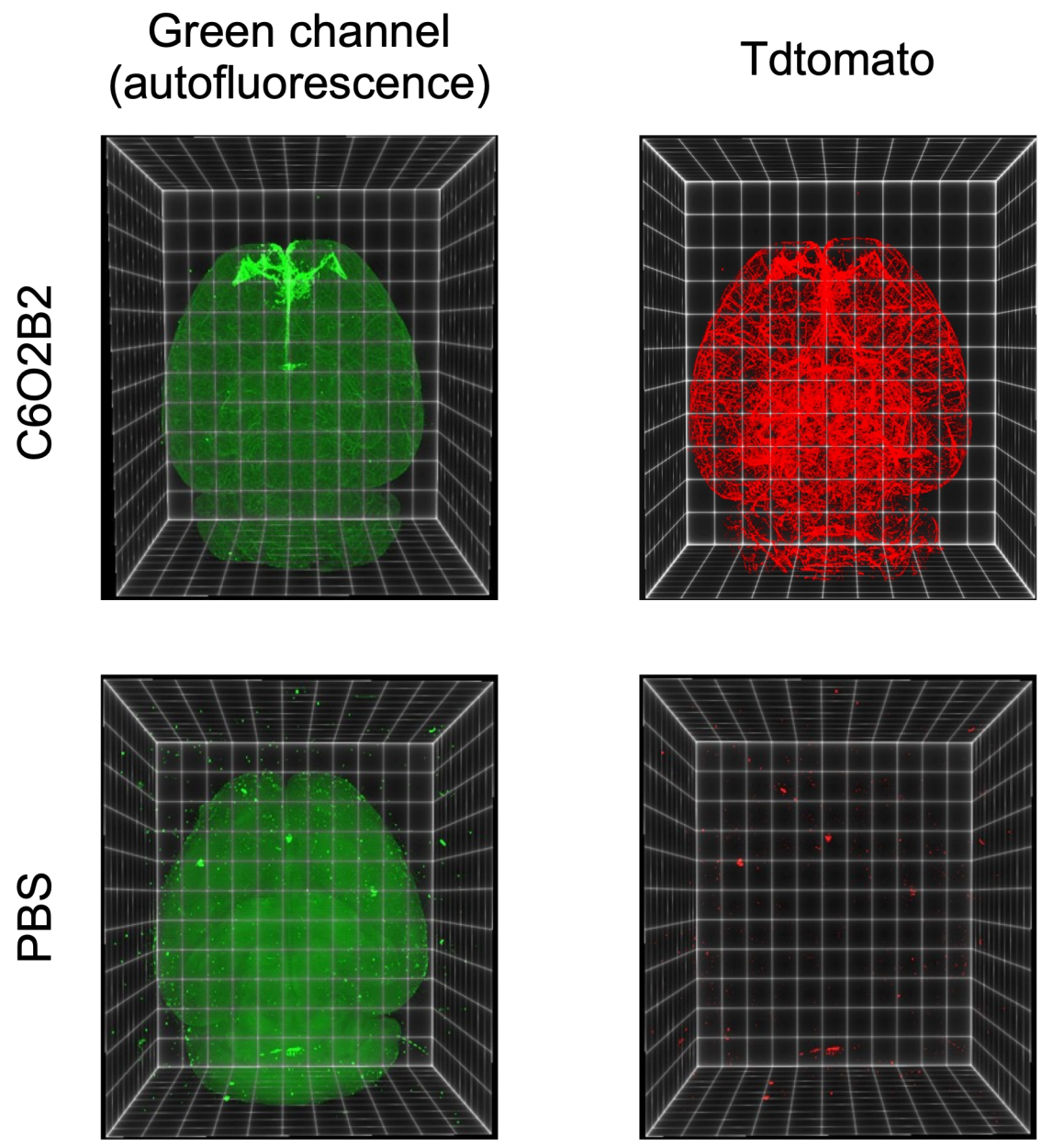

Figure S14. Tissuecyte images of whole brains from PBS- and LNP-Cre treated mice.

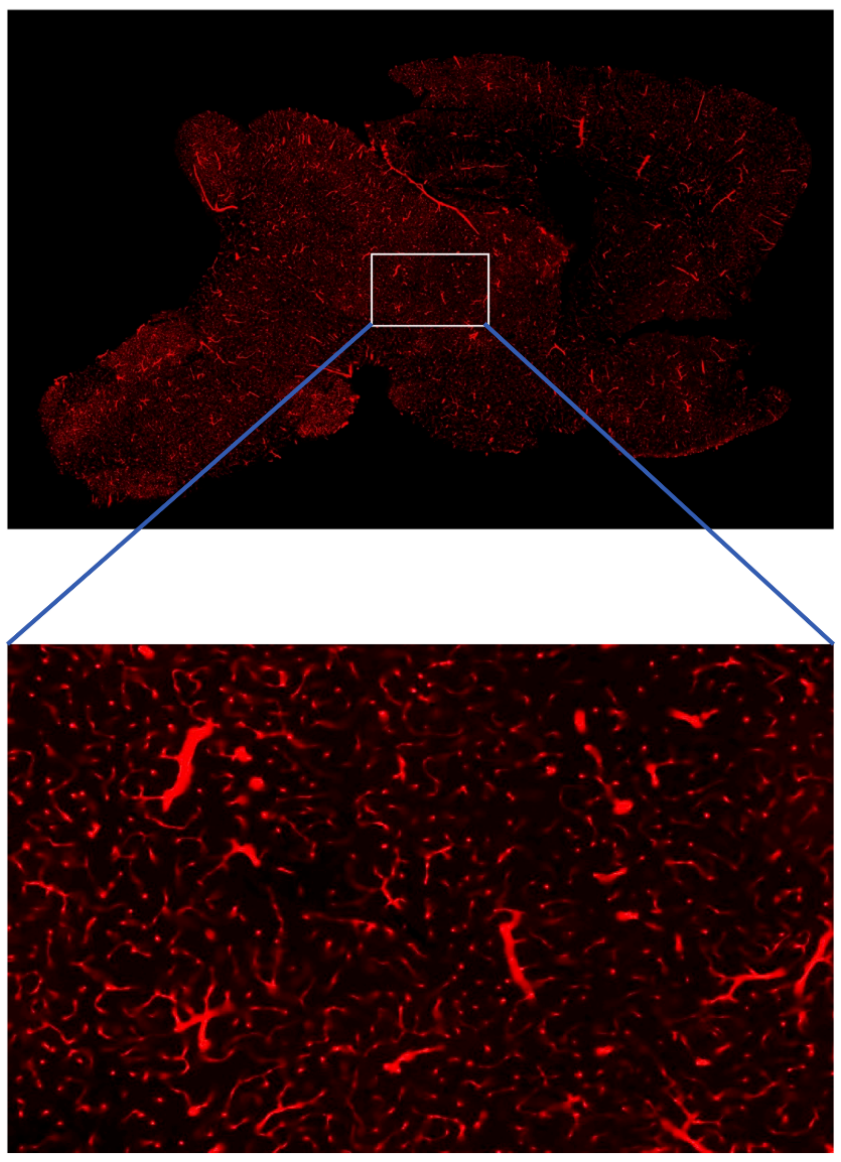

Figure S15. Fluorescence images of brain sections showing tdTomato+ cells in endothelial cells.

Biodistribution and vascular specificity of BBB-targeting LNPs

Cre mRNA was intravenously delivered to Ai14 reporter mice using either BBB-targeting C6O2B2 LNPs or control C6O2 LNPs without the BBB-targeting lipid (C6O2/cholesterol/DODAP/DSPC/DMG-PEG at a molar ratio of 35:35:50:30:2). Transfection across major organs was quantified by flow cytometry. Across total cell populations, BBB-targeting C6O2B2 LNPs did not show significant increased transfection in most peripheral organs, including the liver, lungs, spleen, kidney, stomach, pancreas, and intestine, compared to control C6O2 LNPs. A modest increase was observed in the heart (**Figure S16a**). To further assess vascular specificity, we analyzed endothelial cells ($CD31^+$). BBB-targeting C6O2B2 LNPs resulted in increased endothelial transfection in the heart and pancreas compared to control C6O2 LNPs (**Figure S16b**). Together, these findings demonstrate that while BBB-targeting LNPs can modestly enhance endothelial uptake in select peripheral tissues, their impact on most peripheral organs remains limited.

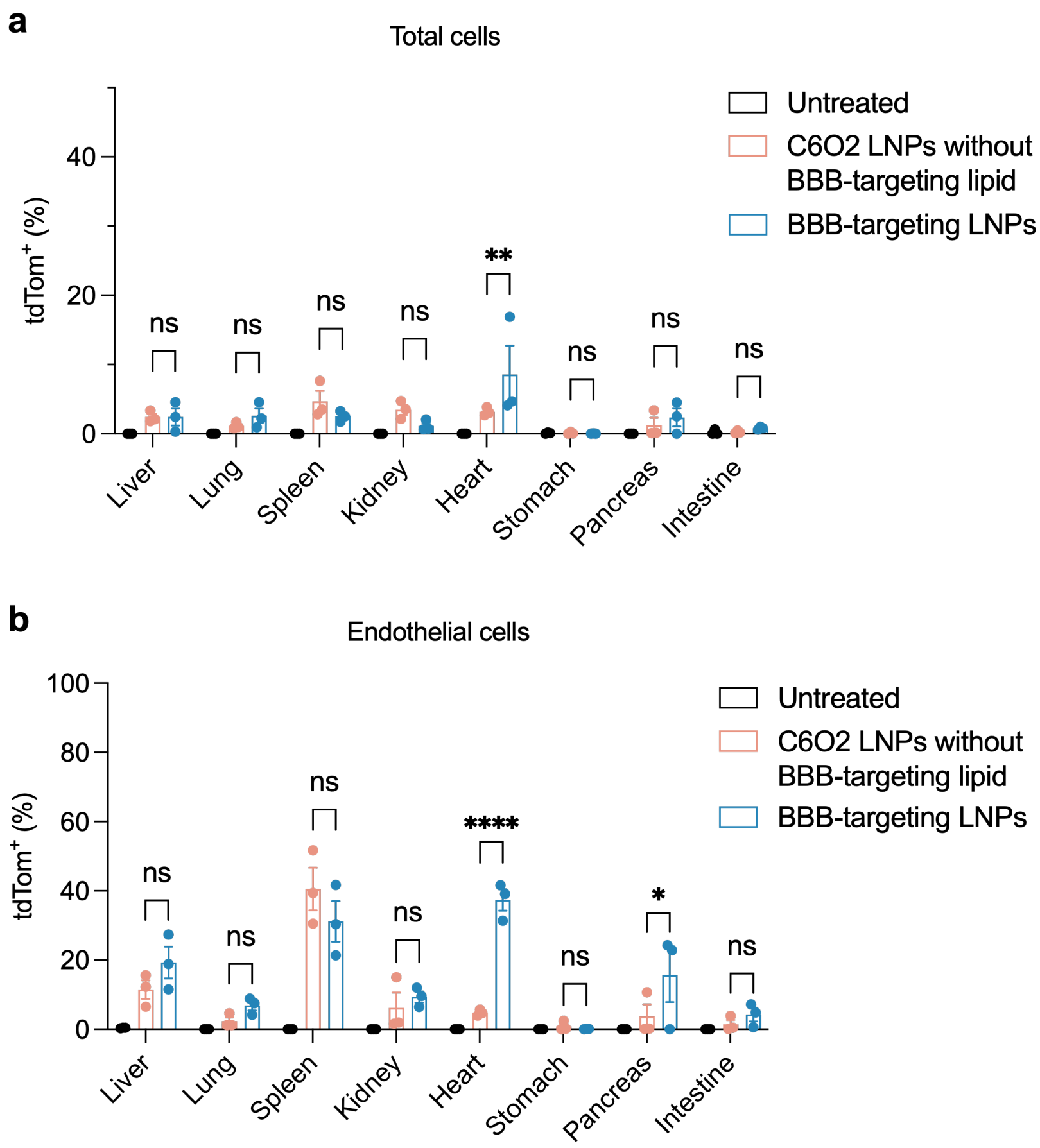

Figure S16. Biodistribution of Cre mRNA delivery across major organs beyond the brain assessed by flow cytometry. (**a**,**b**) BBB-targeting C6O2B2 LNPs or C6O2 LNPs without BBB-targeting lipid encapsulating Cre mRNA were intravenously administered to Ai14 mice (0.3 mg/kg Cre mRNA) on day 0 and 2. On day 4, tdTomato-positive ($tdTom^+$) cells across total cell populations (**a**) and endothelial cells ($CD31^+$) (**b**) in major organs beyond the brain (liver, lungs, spleen, kidney, heart, stomach, pancreas, intestine) were quantified by flow cytometry (n = 3 biologically independent animals). PBS-treated mice served as negative controls. Statistics in (**a**,**b**), two-way ANOVA and Tukey's multiple comparisons test. Data are presented as mean ± s.e.m. $^{*}P < 0.1$, $^{**}P < 0.01$, $^{****}P < 0.0001$, ns: not significant.

Contribution of DODAP and the BBB-Targeting lipid to brain delivery efficiency

To dissect the contribution of individual components, we prepared LNPs with or without DODAP, as well as LNPs lacking the BBB-targeting lipid, and evaluated their performance *in vivo*. Following intravenous delivery of Luc mRNA in C57BL/6 mice, LNPs lacking the BBB-targeting lipid (control C6O2 LNPs: C6O2/cholesterol/DODAP/DSPC/DMG-PEG) showed no detectable expression in the brain. LNPs containing C6O2B2 brain-targeting lipid but lacking DODAP retained the ability to mediate brain delivery, but at reduced efficiency (**Figure S17a**). Incorporation of DODAP increased luciferase expression by approximately 5.6-fold (**Figure S17b**), indicating its role in enhancing transfection efficiency. We further evaluated these formulations by delivering Cre mRNA in Ai14 reporter mice. Consistently, inclusion of DODAP resulted in more than a 2-fold increase in transfection of brain endothelial cells. However, LNPs lacking the BBB-targeting lipid (C6O2 LNPs) showed negligible transfection even formulated with DODAP (**Figure S17c**). Together, these results demonstrate that the BBB-targeting lipid C6O2B2 is essential for enabling brain targeting, whereas DODAP is not required for targeting per se, but plays a critical role in enhancing delivery efficiency.

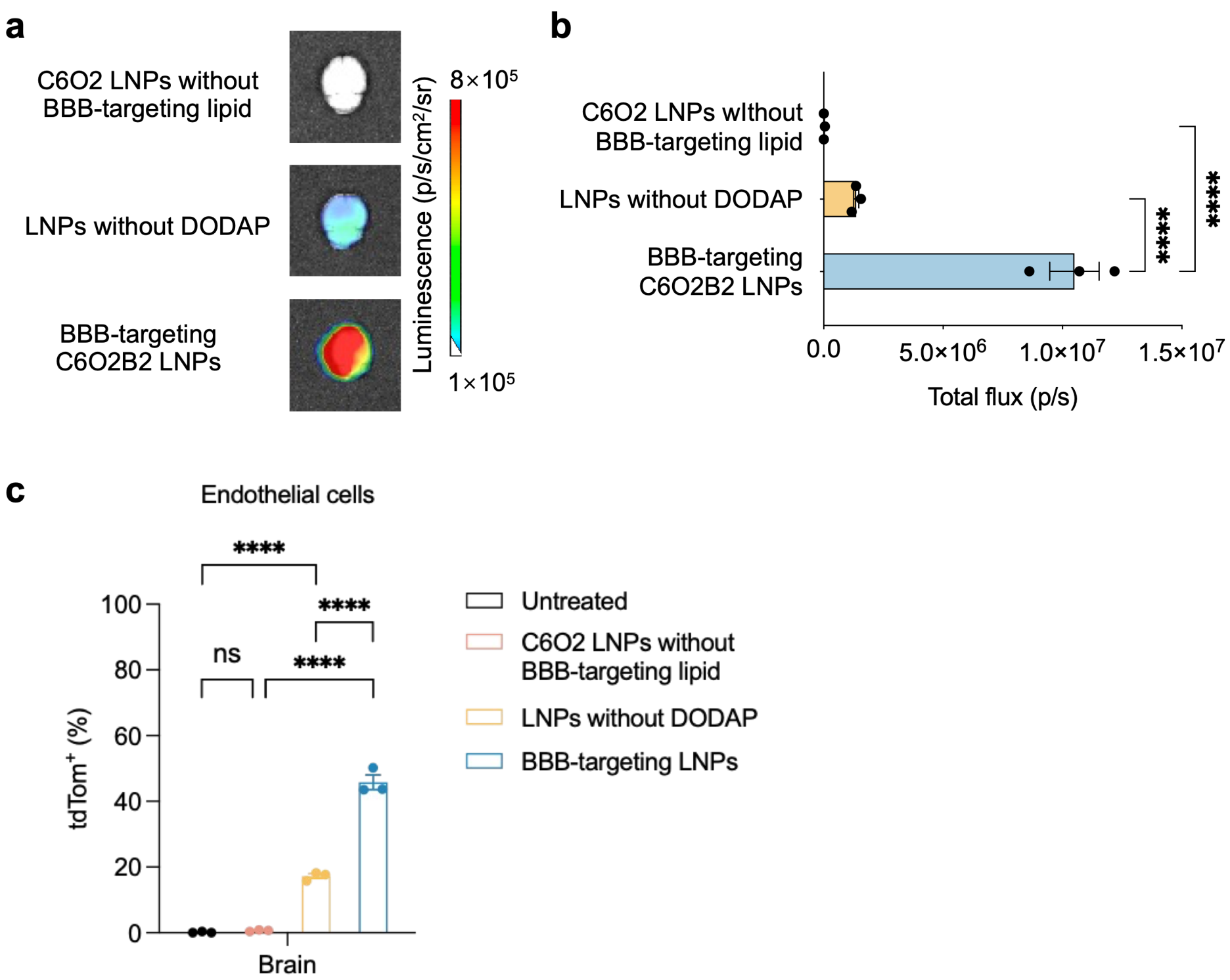

Figure S17. Role of BBB-targeting lipid C6O2B2 and DODAP in impacting BBB-targeting performance. (**a**) Bioluminescence imaging (BLI) was performed 6 h after intravenous administration of Luc mRNA-loaded LNPs (0.1 mg/kg mRNA). Formulations include LNPs lacking the BBB-targeting lipid (C6O2 LNPs), LNPs without DODAP, and BBB-targeting C6O2B2 LNPs. (**b**) Quantification of total brain luminescence flux (n = 3 biologically independent animals). (**c**) BBB-targeting C6O2B2 LNPs, C6O2 LNPs without BBB-targeting lipid, or C6O2B2 LNPs without DODAP encapsulating Cre mRNA were intravenously administered to Ai14 mice (0.3 mg/kg Cre mRNA) on day 0 and 2. On day 4, tdTomato-positive (tdTom+) cells within endothelial cells ($CD31^+$) were quantified by flow cytometry (n = 3 biologically independent animals). PBS-treated mice served as negative controls. Statistics in (**b**,**c**), one-way ANOVA. Data are presented as mean ± s.e.m. $^{****}P < 0.0001$, ns: not significant.

Therapeutic evaluation of IL-10 mRNA in an acute brain Injury model

Disruption of the blood-brain barrier (BBB) during systemic inflammation contributes to the progression of multiple central nervous system disorders. Lipopolysaccharide (LPS) is widely used to model inflammation-induced BBB dysfunction and acute brain injury.[1,2] Interleukin-10 (IL-10), a key anti-inflammatory cytokine, suppresses excessive inflammatory responses and mitigates secondary damage. Given the high transfection efficiency of C6O2B2 LNPs in brain endothelial cells, we evaluated the therapeutic potential of our BBB-targeting platform by delivering IL-10 mRNA in an LPS-induced inflammation model in both prophylactic and therapeutic settings.

<u>Prophylactic setting.</u> To determine whether pre-established IL-10 expression in brain endothelial cells could protect against an inflammatory challenge, C57BL/6 mice were intravenously administered SM-102, C6O2, or C6O2B2 LNPs encapsulating IL-10 mRNA (0.5 mg/kg) at 24 and 3 hours prior to intraperitoneal LPS injection (1.2 mg). Evans blue dye was intravenously injected 6 hours after LPS challenge, and tissues were collected 2 h later for assessment of BBB integrity and inflammatory markers (**Figure S18a**). LPS challenge resulted in pronounced Evans blue accumulation, indicating compromised BBB integrity. However, mice treated with IL-10 mRNA-loaded C6O2B2 LNPs exhibited markedly reduced dye extravasation compared to untreated controls, indicating improved vascular integrity (**Figure S18b**). In addition, IL-10 mRNA level was greatly elevated in LNP treated mice (**Figure S18c**). This was accompanied by a reduction in the mRNA levels of pro- inflammatory cytokines, including TNF-α, IL-1β, and IL-6 (**Figure S18d**-**f**), compared to untreated group, indicating attenuation of the inflammatory response.

<u>Therapeutic setting.</u> C57BL/6 mice were intravenously administered SM-102, C6O2, or C6O2B2 LNPs encapsulating IL-10 mRNA (0.5 mg/kg) at 0.5 and 6 hours after intraperitoneal LPS injection (1.0 mg). Evans blue dye was intravenously injected 12 hours after LPS challenge, and tissues were collected 2 h later for assessment of BBB integrity and inflammatory markers (**Figure S18g**). Despite treatment being initiated after the onset of inflammation, C6O2B2 BBB-targeting LNPs significantly reduced Evans Blue extravasation compared with both SM-102 and C6O2 control formulations, indicating improved BBB integrity (**Figure S18h**). In addition, IL-10 mRNA expression was markedly elevated in mice treated with C6O2B2 LNPs (**Figure S18i**). Consistent with these findings, C6O2B2 LNPs produced the greatest suppression of pro-inflammatory cytokines, including TNF-α, IL-1β, and IL-6, among all treatment groups (**Figure S18j**-**l**).

Together, these results demonstrate that BBB-targeted delivery of IL-10 mRNA can modulate neuroinflammation and restore BBB integrity in acute brain injury.

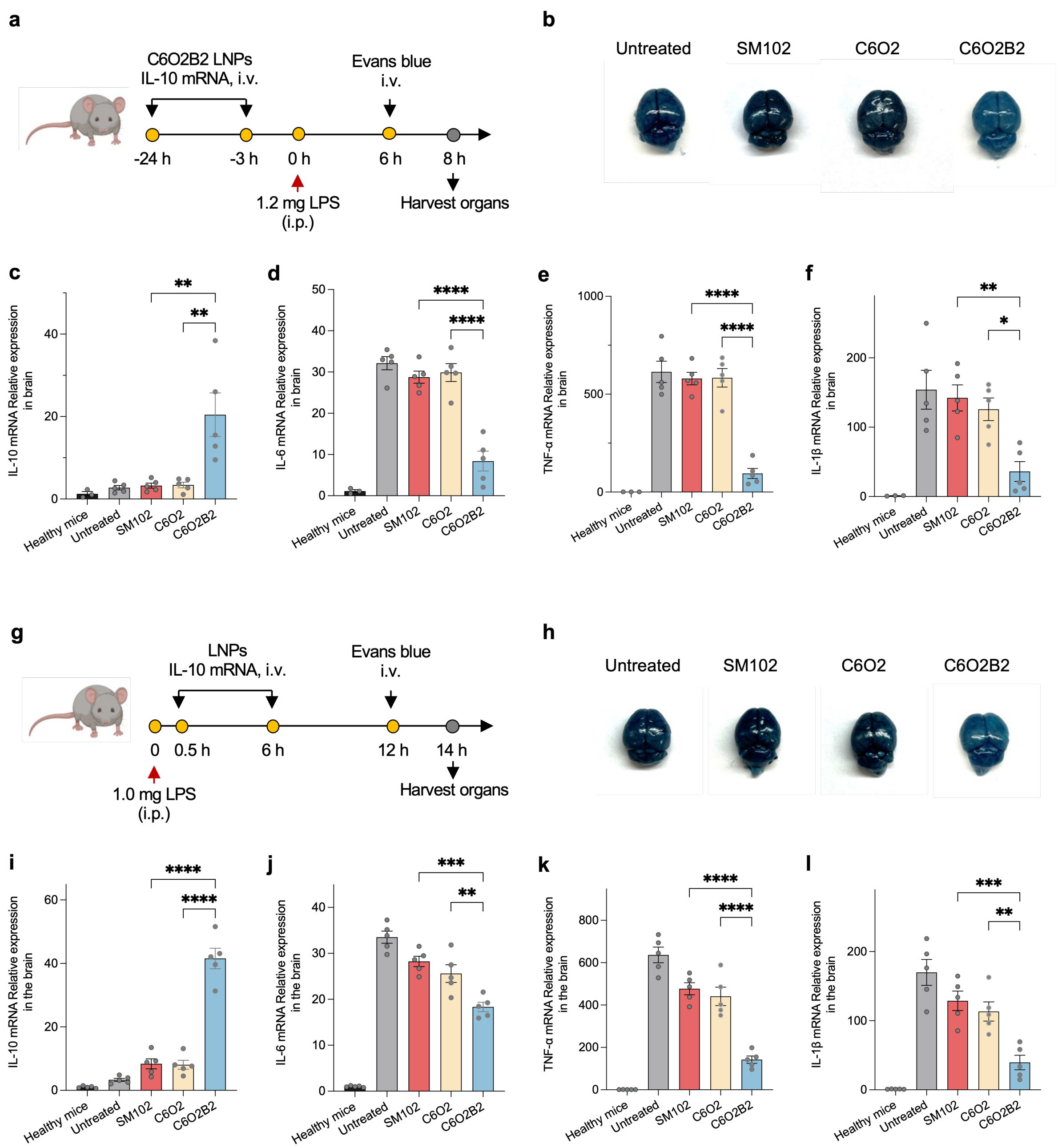

Figure S18. Therapeutic evaluation of BBB-targeted IL-10 mRNA delivery in an LPS-induced acute brain injury model in both prophylactic and therapeutic settings. (**a-f**) Prophylactic setting. Six- to eight-week-old C57BL/6 mice were intravenously administered C6O2B2, SM-102, or C6O2 LNPs encapsulating IL-10 mRNA (0.5 mg/kg) at 24 and 3 hours prior to intraperitoneal LPS injection (1.2 mg). 2% Evans blue dye was administered intravenously 6 hours after LPS challenge, and brains were harvested 2 h later to assess BBB integrity (**a**). Images of brains following Evans blue injection (**b**). IL-10 mRNA levels in brain tissues quantified by qPCR and normalized to Actin (**c**). mRNA levels of IL-6 (**d**), TNF-α (**e**), and IL-1β (**f**) in brain homogenates measured by qPCR. (**g-l**) Therapeutic setting. Six- to eight-week-old C57BL/6 mice were intravenously administered C6O2B2, SM-102, or C6O2 LNPs encapsulating IL-10 mRNA (0.5 mg/kg) at 0.5 and 6 hours after intraperitoneal LPS injection (1.0 mg). 2% Evans blue dye was administered intravenously 12 hours after LPS challenge, and brains were harvested 2 h later to assess BBB integrity (**g**). Images of brains following Evans blue injection (**h**). IL-10 mRNA levels in brain tissues quantified by qPCR and

normalized to Actin (**i**). mRNA levels of IL-6 (**j**), TNF-α (**k**), and IL-1β (**l**) in brain homogenates measured by qPCR. Statistics in (**c**-**f**) and (**i**-**l**), one-way ANOVA and Tukey's multiple comparisons test. Data are presented as mean ± s.e.m. $^{*}P < 0.05$, $^{**}P < 0.01$, $^{***}P < 0.001$, $^{****}P < 0.0001$.

Table S1. Formulations of the top 14 LNPs.

	Molar ratios				Molar percentages (%)				
	C6O2B2	DMPC	PEG	DODAP	C6O2B2	DMPC	PEG	DODAP	Total lipid/mRNA (wt/wt)
**B1**	50	10	4	10	67.57	13.51	5.41	13.51	40
**B2**	65	10	4	10	73.03	11.24	4.49	11.24	40
**B3**	65	10	4	30	59.63	9.17	3.67	27.52	40
**B4**	65	10	4	50	50.39	7.75	3.10	38.76	40
**B5**	65	50	4	10	50.39	38.76	3.10	7.75	40

	Molar ratios				Molar percentages (%)				
	C6O2B2	DOPG	PEG	DODAP	C6O2B2	DOPG	PEG	DODAP	Total lipid/mRNA (wt/wt)
**C1**	50	50	1	50	33.11	33.11	0.66	33.11	40

	Molar ratios				Molar percentages (%)				
	C6O2B2	DOPE	PEG	DODAP	C6O2B2	DOPE	PEG	DODAP	Total lipid/mRNA (wt/wt)
**D1**	35	50	1	50	25.74	36.76	0.74	36.76	40

	Molar ratios				Molar percentages (%)				
	C6O2B2	DSPC	PEG	DODAP	C6O2B2	DSPC	PEG	DODAP	Total lipid/mRNA (wt/wt)
**F1**	35	30	1	50	30.17	25.86	0.86	43.10	40
**F2**	35	30	2	30	36.08	30.93	2.06	30.93	40
**F3**	35	30	2	50	29.91	25.64	1.71	42.74	40
**F4**	50	30	1	50	38.17	22.90	0.76	38.17	40
**F5**	50	30	2	50	37.88	22.73	1.52	37.88	40
**F6**	35	50	1	30	30.17	43.10	0.86	25.86	40
**F7**	65	50	2	50	38.92	29.94	1.20	29.94	40

Table S2. List of mouse primers used for qPCR analysis.

Gene	Forward primer	Reverse Primer
Mouse IL-10	5’- GCT CTT ACT GAC TGG CAT GAG -3’	5’- CGC AGC TCT AGG AGC ATG TG -3’
Mouse Il-6	5’- TAG TCC TTC CTA CCC CAA TTT CC -3’	5’- TTG GTC CTT AGC CAC TCC TTC -3’
Mouse Tnf-α	5’- CTG AAC TTC GGG GTG ATC GG-3’	5’- GGC TTG TCA CTC GAA TTT TGA GA -3’
Mouse IL-1β	5’- GCA ACT GTT CCT GAA CTC AAC T -3’	5’- ATC TTT TGG GGT CCG TCA ACT -3’
Actin	5'-TTT CCA GCC TTC CTT CTT G-3'	5'-GCA TAG AGG TCT TTA CGG ATG-3'

References

[1] W. Liu, Z. Wang, Y. Wang, L. Tong, Y. Li, X. Wei, P. Luan, L. Li, *CNS Neurosci Ther* **2015**, *21*, 568.

[2] C. Wei, W. Jiang, R. Wang, H. Zhong, H. He, X. Gao, S. Zhong, F. Yu, Q. Guo, L. Zhang, L. D. J. Schiffelers, B. Zhou, M. Trepel, F. I. Schmidt, M. Luo, F. Shao, *Nature* **2024**, *629*, 893.
